# Disubstituted 4-Chloro-3-nitrophenylthiourea Derivatives: Antimicrobial and Cytotoxic Studies

**DOI:** 10.3390/molecules23102428

**Published:** 2018-09-21

**Authors:** Anna Bielenica, Giuseppina Sanna, Silvia Madeddu, Gabriele Giliberti, Joanna Stefańska, Anna E. Kozioł, Oleksandra Savchenko, Paulina Strzyga-Łach, Alicja Chrzanowska, Grażyna Kubiak-Tomaszewska, Marta Struga

**Affiliations:** 1 Department of Biochemistry, Medical University, 02-097 Warszawa, Poland; paulina.strzyga@gmail.com (P.S.-Ł.); chrzanow.a@gmail.com (A.C.); mstruga@wum.edu.pl (M.S.); 2 Department of Biomedical Sciences, Section of Microbiology and Virology, University of Cagliary, Cittadella Universitaria, 09042 Monserrato, Italy; g.sanna@unica.it (G.S.); silvia.madeddu@unica.it (S.M.); ggiliberti@unica.it (G.G.); 3 Department of Pharmaceutical Microbiology, Medical University, 02-007 Warszawa, Poland; jstefanska@wum.edu.pl; 4 Faculty of Chemistry, Maria Curie-Sklodowska University, 20-031 Lublin, Poland; anna.koziol@poczta.umcs.lublin.pl; 5 Ecotech Complex, Maria Curie-Sklodowska University, 20-031 Lublin, Poland; oleksandra.savchenko@poczta.umcs.lublin.pl; 6 Department of Biochemistry and Pharmacogenomics, Faculty of Pharmacy, Medical University of Warsaw, 02-097 Warszawa, Poland; grazynakubiak@poczta.onet.pl; 7 Laboratory of Centre for Preclinical Research, Medical University of Warsaw, 02-097 Warszawa, Poland

**Keywords:** antimicrobial activity, biofilm, cytotoxicity, thiourea derivatives, X-ray crystallography

## Abstract

4-Chloro-3-nitrophenylthioureas **1**–**30** were synthesized and tested for their antimicrobial and cytotoxic activities. Compounds exhibited high to moderate antistaphylococcal activity against both standard and clinical strains (MIC values 2–64 μg/mL). Among them derivatives with electron-donating alkyl substituents at the phenyl ring were the most promising. Moreover, compounds **1**–**6** and **8**–**19** were cytotoxic against MT-4 cells and various other cell lines derived from human hematological tumors (CC_50_ ≤ 10 μM). The influence of derivatives **11**, **13** and **25** on viability, mortality and the growth rate of immortalized human keratinocytes (HaCaT) was observed.

## 1. Introduction

In the field of medical chemistry, thiourea derivatives are of great interest due to their synthetic utility and biological importance. The thiourea moiety is a structural part of many bioactive compounds, that display antimicrobial [1,2,3,4,5], antifungal [6], antitubercular [2,7,8], antiviral [9,10,11,12], and central nervous system stimulating [13,14,15,16] properties. Derivatives of this group are also viewed as one of the most promising classes of anticancer agents. 1,2,4-Triazole-linked (thio)urea conjugates have been described as cytotoxic and apoptosis inducing substances [17]. The same properties, supported by DNA topoisomerase II inhibition, were proved for podophylltoxin-thiourea congeners [18]. Diaryl thiourea derivatives bearing 1H-indazole-3-amine scaffold displayed anti-angiogenic potential as selective multi-target receptor tyrosine kinase (RTK) inhibitors [19]. Particularly, they have blocked activities of VEGFR-2, Tie-2 and EphB4 kinases, thus as a result can prevent tumorigenesis. On the other hand, bis-thiazoles derived from disubstituted thioureas are supposed to be involved in the regulation of the expression of acetylcholinesterase (AChE) and butyrylcholinesterase (BuChE), overexpressed in pathological conditions; e.g., cancer [20]. Another group, thioureido benzenesulfonamide derivatives, was found to inhibit carbonic anhydrase CA IX, which triggers the migration of tumor cells and results in a raise of the aggressive/invasive phenotype of cancer [6]. Pyrimidinyl acyl thioureas have shown the ability to block Heat Shock Protein 90 (Hsp90) ATP binding site [21]. The role of this protein is stabilization and regulation of oncogenic client proteins, therefore designing its inhibitors is a novel cancer therapy method. What is more, in different types of cancer, overexpression and mutation of the epidermal growth factor receptor (EGFR) have been observed; thus, finding inhibitors of EGFR kinases among (thio)ureido-quinazoline derivatives is also a promising approach to tumor treatment [22,23]. It was proven that the anticancer potency of symmetrical 1,3-phenyl bis-thiourea compounds were combined with their tubulin-binding properties [24]. By halting mitosis of tumor cells in prometaphase, this lead to their death via apoptosis. A series of pyrimidine-thiourea conjugates were found to exhibit an inhibitory effect against histone lysine demethylase 1 (LSD1), overexpressed in gastric, prostate, lung, and breast cancer cells [25,26]. Moreover, miscellaneous thiourea derivatives carrying sulfonamide moieties were proven to possess antiproliferative properties by docking to the active site of the mitogen kinase enzyme (MK-2), controlling the signal transduction pathway in cell proliferation [5,27,28]. The cytotoxicity of acridinylthioureas originates from their ability to intercalate with DNA and as a result inhibit telomerase or topoisomerase action [29]. Thiourea-derived compounds were also employed in the field of coordination chemistry as versatile chelating agents. The Ru(II), Pd(II) and Pt(II)-based complexes of N,N-disubstituted thioureas have exerted cytotoxic properties via DNA binding ability [30,31,32].

A noticeable cytotoxic potential of mentioned thiourea-derived compounds was observed against a wide range of solid tumors, such as breast [4,6,17,21,23,32,33], lung [4,20,22,23,27,29,31,34], liver [5], prostate [4,18,31,32,33], cervical [4,29,30], brain [20,24,29], gastric [25], or colon [27,29,35] carcinoma, as well as human leukemia [29]. Simultaneously some of them, when tested on normal human cells, were found to possess a low cytotoxicity profile [1,17,20]. The main structural element of anticancer active thioureas were the presence of “the butterfly-like” tricyclic ring of acridine [29] or other heterocycles, such as pyrimidine [5,6,21], quinoline [27], quinazoline [22,23], furane [32], benzimidazole [4], chrysene [33], benzodioxole [18], triazole [17] or thiazole [20]. On the other hand, the most promising functionalities of a terminal aromatic ring were mainly electron-withdrawing substituents, such as nitro [27,33,35], cyano [17], trifluoromethyl/methoxy [17,19], halogeno [5,20,22,23], but also electron-withdrawing—methoxy [18,21,25] and methyl [24] groups. Among them, compounds containing the nitro group attached to the aromatic moiety (e.g., nitrofurans, bisnaphthalimides) are typically used as antibiotics and antimicrobials [36,37]. The introduction of an additional deactivating substituent, such as a halogen atom, might increase the bioactivity of a disubtituted derivative, because of a stronger electronegativity effect achieved [1,2].

Prompted by the above facts and as a continuation of our interest in biologically active thioureas [1,2,38], we report the synthesis of some new 4-chloro-3-nitrophenylthiourea derivatives and complete the cytotoxic profile of compounds of this group [2].

## 2. Results and Discussion

### 2.1. Chemistry

**Scheme 1:**
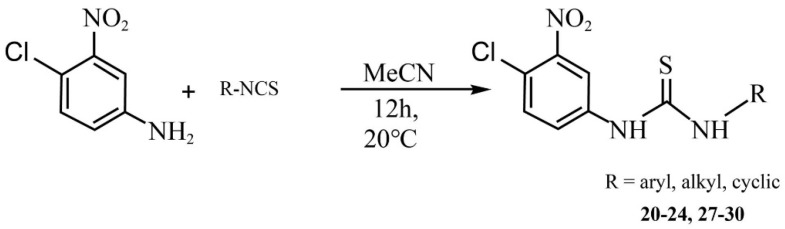
Synthesis of 4-chloro-3-nitrophenylthiourea derivatives **20**–**24**, **27**–**30**.

In this paper new 1,3-disubstituted thioureas **20**–**24** and **27**–**30** were synthesized from 4-chloro-3-nitroaniline and variety of isothiocyanates (Scheme 1, Table 1). Reactions were conducted in anhydrous acetonitrile at room temperature. The structures of new compounds were established by spectroscopic methods (^1^H NMR, ^13^C NMR, MS, and IR). The derivatives **1**–**19** and **25**–**26** were obtained previously [2].

### 2.2. Antimicrobial Studies

Newly obtained 4-chloro-3-nitrophenylthioureas **20**–**24** and **27**–**30** were evaluated for their in vitro antimicrobial activity against a set of bacterial and fungal strains. The initial testing by the disc diffusion method [39,40] chose derivatives with meaningful antimicrobial properties. Considering the fact that all compounds exhibited activity against Gram-positive strains (GIZ ≥ 13 mm), in the next step the minimal inhibitory concentrations (MIC) were assessed by the twofold serial microdilution method [41,42]. The results showed that all investigated compounds (except **21**) exhibited the potential to moderate antibacterial potency, mainly against standard staphylococcal and *M. luteus* strains (Table 2).

A close examination of the structures of the active compounds revealed that the nature of the substituent at the thiourea moiety governs their antimicrobial potential. In general, the most prominent activity against standard strains was confined to derivatives comprising the electron-donating alkyl groups at the C2/C4 position of the aromatic ring (**20**, **22**). For these derivatives the MIC values ranged from 2 to 4 μg/mL. The moderate activity (MIC range 8–16 μg/mL) towards staphylococci was denoted for bicyclo[2.2.1]hept-2-yl- (**24**), phenyl- (**23**), furan-2-ylmethyl- (**30**) and (thio)alkylthioureas (**27**, **29**). What is more, compounds possessing an alkyl chain at the thiourea nitrogen (**27**–**29**) exerted some activity against *P. vulgaris*, representing Gram-negative strains (MIC 32–128 μg/mL). Only one analog of the series, 4-methoxyphenylthiourea (**21**), was devoid of activity against tested microorganisms.

The potency of thioureas **22** and **24** towards all hospital methicillin-resistant strains of *S. epidermidis* was 2–16 stronger than the reference Ciprofloxacin (Table 3). The antistaphylococcal activity of other tested compounds was equal or 2–4 times higher than observed for the standard antibiotic. Selected strains (MRSE *405/11*, *406/11*, *409/11*, *411/11*, *422/11*) were remarkably less susceptible for the presence of synthesized thiourea than for the reference drug.

**Figure 1:**
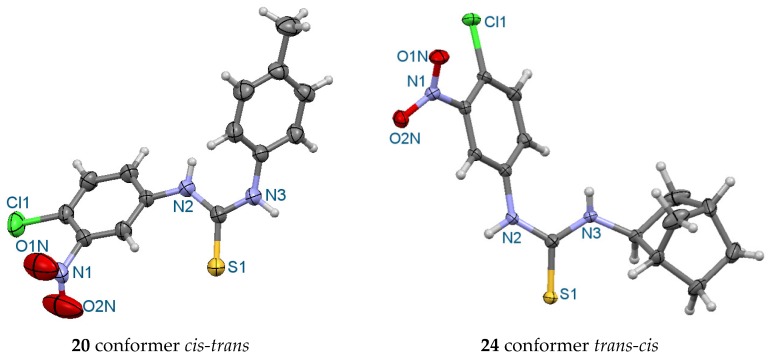
Perspective view of molecules **20** and **24**. Conformers are defined by relative orientation of 4-chloro-3-nitrophenyl and aryl/alkyl groups to thiourea C=S bond.

It is worth noting that the factor influencing the biological activity could be a molecular flexibility. It is evident from the determined crystal structures of **20** and **24** that molecules of this group of derivatives are able to adopt different conformations (Figure 1). Variable orientations of the thiourea C=S bond versus either 4-chloro-3-nitrophenyl or the second substituent could be described as *cis*-*trans* and *trans*-*cis*, respectively. Further, the patterns of intermolecular hydrogen bonding are specific for given conformers. 

### 2.3. Cytotoxicity and Anti-HIV Activity

Due to the previously reported anti-HIV activities of thiourea derivatives [11,43], both formerly (**1**–**19**, **25**, **26**) and newly synthesized compounds (**20**–**24**, **27**–**30**) were tested in cell-based assay against the human immunodeficiency virus type-1 (HIV-1), using Efavirenz as the reference inhibitor. The cytotoxicity against MT-4 cells was evaluated in parallel with the antiviral activity (Table 1).

None of tested compounds showed selective antiviral activity against HIV-1. However, all compounds turned out interestingly cytotoxic for exponentially growing MT-4 cells, with many of them cytotoxic in the low micromolar range (CC_50_ < 10 µM). The antiproliferative activity against the CD4+ human T cell line derived from a hematological human tumor prompted us to evaluate the antiproliferative activity of a group of cytotoxic compounds (**1**, **4**, **6**, **9**, **10**, **11**, **13**, **14**, **17**, **19**), also for a panel of other human and solid tumors, as well as for cell lines derived from normal human tissues (Table 4).

Interestingly, the tested compounds showed CC_50_ values comparable to that obtained with MT-4 cells, but they did not result highly selective, showing cytotoxicity also against the “normal” CRL7065 cell lines, even if with lower values. The highest selectivity versus CCRF-CEM and SK-MEL-28 cells was observed for 4-bromophenyl (**6**) and 4-iodophenylthiourea (**17**) derivatives. None of the tested compounds showed CC_50_ values comparable with Camptotecin, used as the reference compound.

### 2.4. Cytotoxic Activity in HaCaT Cells

The most promising antistaphylococcal (**11**, **13**) and antimycobacterial (**25**) agents among derivatives presented in our previous study [2] were now screened with respect to their toxicity against HaCaT cell line (health cell line from adult human skin). The HaCaT cell line was chosen, because it exhibits normal differentiation capacity, and its DNA fingerprint pattern is unaffected by long-term cultivations, transformations, and multiple chromosomal alterations [44].

**Figure 2:**
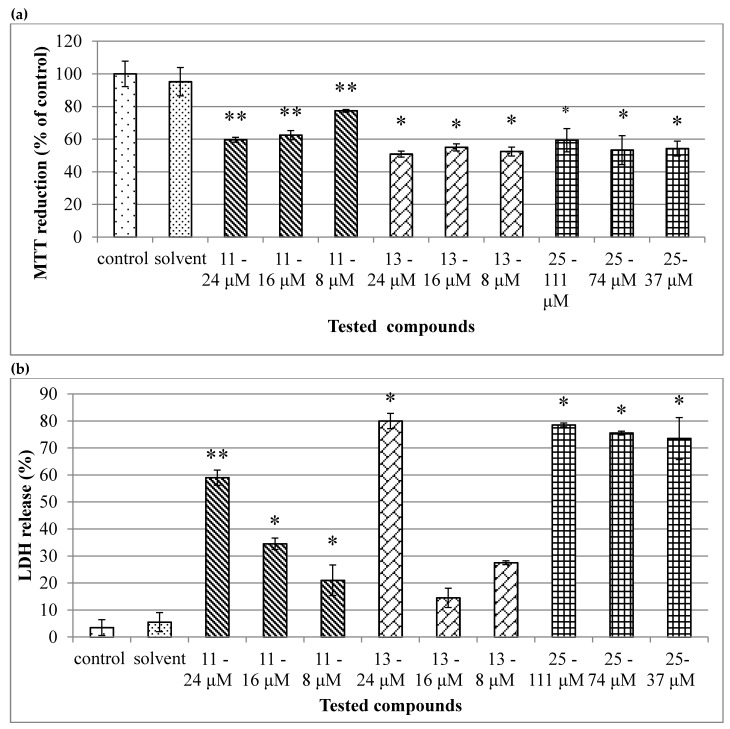
Cell viability assessed by MTT mitochondrial reduction (**a**), and LDH release (**b**) as a marker of cell death in HaCaT cells treated for 24 h with compounds **11**, **13** and **25**. Data are expressed as means ± SD from three independent experiments performed in triplicate. Statistical significance: * *p* < 0.01 and ** *p* < 0.001 refer to the control (untreated) cells.

Human keratinocyte cell lines were exposed to different concentrations of synthesized compounds for 24 h. Derivatives were tested in concentrations equal to their cytotoxic concentrations against MT-4 cells (i.e., 8 µM for **11** and **13**, and 37 µM for **25**), as well as in concentrations 2 and 3 times higher. At the concentration 8 µM, the thiourea compound **11** caused approximately 20% decrease in cell viability, in comparison with controlled and untreated cells (Figure 2a), that indicated its weak cytotoxic influence against normal cells. On the other hand, cytotoxicity of antibacterial agents **13** and **25** observed at the level of 50%, was found to be moderate according to ISO 10993-5 standards [45].

Results of the LDH (lactate dehydrogenase release) assay indicated that cell mortality in the presence of compounds **11** and **13** increased to 20% and 30%, respectively (Figure 2b). The LD_10_ value (concentration at which 10% of cells were dead) was determined at the lowest investigated concentration of the compound **11** (8 µM). Substance **13** at concentrations 8 and 16 µM was found to be lowly cytotoxic, resulting in an approximate 10% release of LDH. Compound **25** had the strongest impact on the mortality of tested cells, however, its concentration was the highest among all tested compounds (37 µM). All applied concentrations of that derivative resulted in 7-fold higher cytotoxicity as compared to the LD_10_ value. 

**Figure 3:**
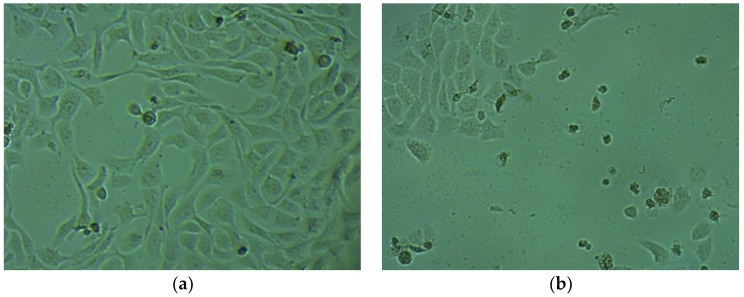

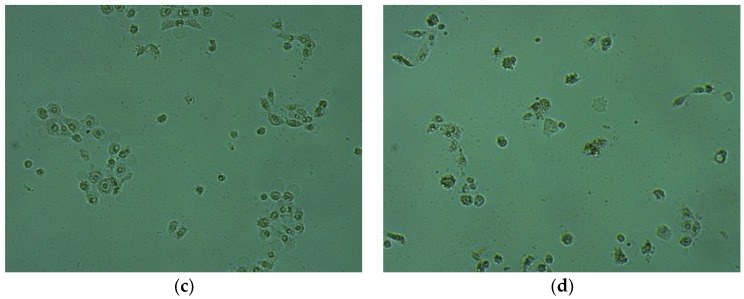
Morphological changes of HaCaT cells after 24 h treatment with compounds **11**, **13** and **25**, observed under the light microscope (800×): Untreated cells (**a**). Cells treated for 24 h with compounds **11**, **13** and **25** in concentrations 8 µM, 8 µM and 37 µM, respectively (**b**–**d**).

**Figure 4:**
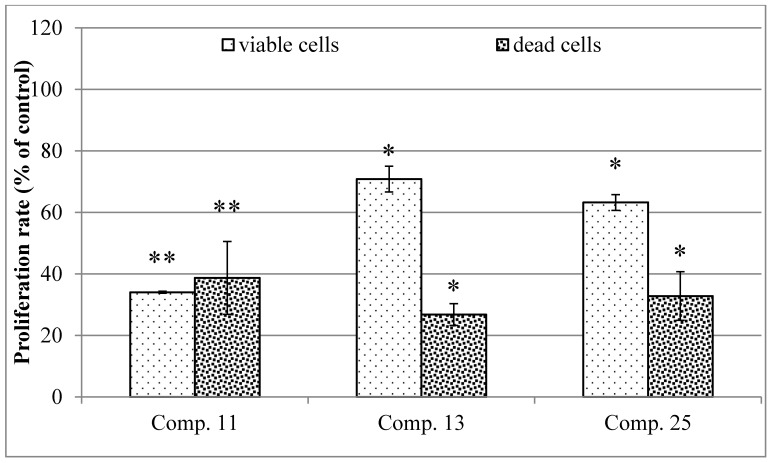
Proliferation rate assessment in HaCaT cells treated for 48 h with compounds **11**, **13** and **25**, applied at concentrations 8 µM, 8 µM and 37 μM, respectively. Data are expressed as means ± SD from three independent experiments performed in triplicate. Statistical significance: * *p* < 0.01 and ** *p* < 0.001 refer to the control (untreated) cells.

Significant concentration-dependent changes in shape, size, and density of HaCaT cells were observed under a light microscope after 24 h treatment with lowest concentrations of all investigated compounds (Figure 3). At given concentrations, derivatives inhibited viable cell proliferation by about 30–40% (compounds **13** and **25**) or 65% (**11**) (Figure 4). This suggests a direct effect on the HaCaT viability, which was confirmed by the MTT assay. Derivatives **13** and **25** have demonstrated an increased proliferation rate.

The tested substances modified cell viability as well as mortality and growth rate of HaCaT cultures. The presented results demonstrate that derivative **11**, one of the strongest antistaphylococcal and antibiofilm agents, at its lowest concentration, possessed the lowest toxic effect on HaCaT cells. Our studies suggest that all tested substances in their lowest concentrations were less toxic in normal immortalized cell lines in comparison to cancer cell lines, however, further studies are needed to describe the underlining mechanism for future application.

## 3. Materials and Methods 

### 3.1. Chemistry

All reagents and solvents were purchased from Alfa Aesar, Sigma Aldrich or POCh (Polskie Odczynniki Chemiczne, Gliwice, Poland). The infrared (IR) spectra were obtained on Perkin Elmer Spectrum 1000 spectrometer. The nuclear magnetic resonance (NMR) spectra were recorded on Varian VNMRS 300 Oxford NMR spectrometer, using tetramethylsilane (TMS) as the internal reference. Mass spectral electrospray ionization (ESI) measurements were carried out on Waters ZQ Micro-mass instruments with quadruple mass analyzer, at a declustering potential of 40–60 V. Flash chromatography was performed on Merck silica gel 60 (200–400 mesh) using chloroform eluent.

General procedure for the synthesis of (4-chloro-3-nitrophenyl)thiourea derivatives (**20**–**24**, **27**–**30**)

To a solution of 4-chloro-3-nitroaniline (0.0029 mol; 0.5g) in dried acetonitrile (10 mL), a corresponding isothiocyanate (0.0029 mol) was added. The mixture was stirred at room temperature for 12 h. Next the solvent was evaporated and the solid residue was either crystallized from acetonitrile (chloroform) or purified by column chromatography (chloroform). 

The synthesis of derivatives **1**–**19**, **25**, **26** was published formerly [2].

1-(4-chloro-3-nitrophenyl)-3-(4-methylphenyl)thiourea (**20**)

Yield 30%, yellow crystals, m.p. 156–158 °C. FT-IR (KBr, cm^−1^): 3326.2, 3196.1 (ν N-H); 3113.6, 3024.4 (ν ar C-H); 2977.6 (ν alC-H); 1589.6 (δ N-H); 1533.8 (ν N-O); 1482.0 (ν arC-C); 1407.0 (δ ar(1,4)C-C); 1358.6 (ν N-O); 1299.9 (δ arC-H); 1236.4 (ν alC-C); 1159.7, 1145.4 (δ arC-H); 1043.3 (ν C=S); 864.0 (δ C-Cl); 703.7 (ν C=S). ^1^H NMR (300 MHz, DMSO) δ: 10.07–10.05 (m, 2H, NH), 8.35 (d, 1H, *J* = 2.7 Hz, H-2), 7.80 (dd, 1H, *J*_1_ = 2.4 Hz, *J*_2_ = 2.7 Hz, H-6), 7.69 (d, 1H, *J* = 8.7 Hz, H-5), 7.34–7.29 (m, 2H, H3′, H-5′), 7.18–7.16 (d, 2H, *J* = 8.1 Hz, H-2′, H-6′), 2.29 (s, 3H, H-4′a). ^13^C NMR (75.4 MHz, DMSO) δ: 179.63 (C=S), 146.78 (C-3), 139.86 (C-1), 136.13 (C-1′), 134.40 (C-4′), 131.20 (C-4), 129.13 (C-3′, C-5′), 128.34 (C-5), 124.11 (C-2′, C-6′), 119.69 (C-2), 119.28 (C-6), 20.52 (C-4′a). ESI MS: *m*/*z* = 320.0 [M − H]^−^ (100%). Rf for TLC (CHCl_3_:MeOH (1%)): 0.12.

Crystal data: crystal system triclinic, space group P-1, unit cell dimensions at 293 K: a = 7.442(1) Å, b = 8.012(2) Å, c = 12.944(3) Å, α = 98.74(3)°, β = 94.81(3)°, γ = 100.17(3)°, V = 746.0(3) Å3, Z = 2, D_calc_ = 1.433 g/cm^3^, F(000) = 332, μ = 3.647 mm^−1^, θ range = 3.48 to 75.15°, reflections collected/independent/observed 3147/3042/957, max. and min. transmission 0.7118 and 0.4283; Goodness-of-fit on F^3^ 0.929, final R indices [I > 2σσI)], R1 = 0.0479, wR2 = 0.1266, R indices (all data), R1 = 0.2455, wR2 = 0.1857, residual electron density max./min. 0.38 and −0.36 e Å^−3^. CCDC No. 1849698.

1-(4-chloro-3-nitrophenyl)-3-(4-methoxyphenyl)thiourea (**21**)

Yield 28%, yellow powder, m.p. 149–150.5 °C. FT-IR (KBr, cm^−1^): 3241.8, 3201.5 (ν N-H); 3056.2 (ν arC-H); 1588.4 (δ N-H); 1548.9 (ν N-O); 1484.8 (ν arC-C); 1405.6 (δ ar(1,4)C-C); 1345.5 (ν N-O); 1241.5 (ν C-O-C); 1175.9, 1125.5 (δ arC-H); 1038.3 (ν C=S); 880.2 (δ C-Cl); 697.2 (ν C=S). ^1^H NMR (300 MHz, DMSO) δ: 9.98 (s, 2H, NH), 8.34 (d, 1H, *J* = 2.4 Hz, H-2), 7.80 (dd, 1H, *J*_1_ = 2.4 Hz, *J*_2_ = 2.7 Hz, H-6), 7.68 (d, 1H, *J* = 8.7 Hz, H-5), 7.34–7.29 (m, 2H, H-3′, H-5′), 6.96–6.91 (m, 2H, H-2′, H-6′), 3.76 (s, 3H, H-4′a). ^13^C NMR (75.4 MHz, DMSO) δ: 179.54 (C=S), 156.65 (C-4′), 146.46 (C-3), 139.63 (C-1), 131.15 (C-1′), 130.88 (C-4), 128.06 (C-5), 125.88 (C-2′, C-6′), 119.41 (C-2), 118.93 (C-6), 113.61 (C-3′, C-5′), 54.97 (C-4′a). ESI MS: *m*/*z* = 336.0 [M − H]^−^ (100%). Rf for TLC (CHCl_3_:MeOH (1%)): 0.17.

1-(4-butyl-2-methylphenyl)-3-(4-chloro-3-nitrophenyl)thiourea (**22**)

Yield 40%, dark yellow solid, m.p. 67–69 °C. FT-IR (KBr, cm^−1^): 3341.6, 3187.5 (ν N-H); 3098.1 (ν arC-H); 2954.5, 2859.9 (ν alC-H); 1584.5 (δ N-H); 1533.7 (ν N-O); 1481.5 (ν arC-C); 1407.5 (δ ar(1,4)C-C); 1342.5 (ν N-O); 1126.3 (δ arC-H); 1045.6 (ν C=S); 872.3 (δ C-Cl); 715.2 (ν C=S). ^1^H NMR (300 MHz, DMSO) δ: 9.95 (s, 1H, NH), 9.66 (s, 1H, NH), 8.36 (d, 1H, *J* = 2.1 Hz, H-2), 7.82 (dd, 1H, *J*_1_ = *J*_2_ = 2.7 Hz, H-6), 7.68 (d, 1H, *J* = 9.0 Hz, H-5), 7.17–6.98 (m, 3H, H-3′, H-5′, H-6′), 2.57–2.55 (m, 2H, H-4a′), 2.20 (s, 3H, H-2′a), 1.58–1.50 (m, 2H, H-4′b), 1.35–1.27 (m, 2H, H-4′c), 0.93–0.87 (m, 3H, H-4′d). ^13^C NMR (75.4 MHz, DMSO) δ: 180.42 (C=S), 146.69 (C-3), 141.03 (C-2′), 140.57 (C-4′), 139.92 (C-1), 134.53 (C-1′), 131.14 (C-4), 130.356 (C-3′), 128.40 (C-5), 127.65 (C-5′), 126.16 (C-6′), 119.74 (C-2), 119.26 (C-6), 34.35 (C-4′a), 33.04 (C-4′b), 21.77 (C-4′c), 17.75 (C-2′a), 13.76 (C-4′d). ESI MS: *m*/*z* = 376.1 [M − H]^−^ (100%). Rf for TLC (CHCl_3_:MeOH (1%)): 0.08.

1-(4-chloro-3-nitrophenyl)-3-phenylthiourea (**23**)

Yield 56%, pale yellow powder, m.p. 163–165 °C. FT-IR (KBr, cm^−1^): 3323.5, 3161.3 (ν N-H); 3089.6, 2982.8 (ν arC-H); 1592.0 (δ N-H); 1527.6 (ν N-O); 1482.0 (ν arC-C); 1407.5 (δ ar(1,4)C-C); 1362.7 (ν N-O); 1298.8, 1187.6, 1143.4 (δ arC-H); 1047.1 (ν C=S); 863.7 (δ C-Cl); 695.2 (ν C=S). ^1^H NMR (300 MHz, DMSO) δ: 10.15 (s, 2H, NH), 8.35 (d, 1H, *J* = 2.4 Hz, H-2), 7.81 (dd, 1H, *J*_1_ = *J*_2_ = 2.4 Hz, H-6), 7.70 (d, 1H, *J* = 9.0 Hz, H-5), 7.47–7.44 (m, 2H, H-2′, H-6′), 7.37 (t, 2H, *J* = 7.8 Hz, H-3′, H-5′), 7.18 (t, 1H, *J* = 7.35 Hz, H-4′). ^13^C NMR (75.4 MHz, DMSO) δ: 179.95 (C=S), 147.09 (C-3), 140.05 (C-1′), 139.08 (C-1), 131.53 (C-4), 128.92 (C-3′, C-5′), 128.62 (C-5), 125.31 (C-4′), 124.20 (C-2′, C-6′), 119.98 (C-2), 119.65 (C-6). ESI MS: *m*/*z* = 330.0 [M + Na]^+^ (100%). Rf for TLC (CHCl_3_:MeOH (1%)): 0.15.

1-bicyclo[2.2.1]hept-2-yl-3-(4-chloro-3-nitrophenyl)thiourea (**24**)

Yield 30%, dark yellow solid, m.p. 94–96 °C. FT-IR (KBr, cm^−1^): 3304.1, 3196.0 (ν N-H); 3033.5 (ν arC-H); 2953.0, 2869.5 (ν alC-H); 1576.1 (δ N-H); 1540.6 (ν N-O); 1478.9 (ν arC-C); 1408.6 (δ ar(1,4)C-C); 1343.4 (ν N-O); 1258.5, 1133.8, 1109.6 (δ arC-H); 1047.2 (ν C=S); 883.3 (δ C-Cl); 714.7 (ν C=S). ^1^H NMR (300 MHz, DMSO) δ: 9.65 (s, 1H, NH), 8.47 (s, 1H, NH), 8.06 (m, 1H, H-2), 7.74 (dd, 1H, *J*_1_ = *J*_2_ = 2.4 Hz, H-6), 7.65 (d, 1H, *J* = 9.0 Hz, H-5), 3.98 (br. s, 1H, H-1′), 2.29–2.26 (m, 2H, H-7′), 1.77–1.70 (m, 1H, H-2′), 1.54–1.30 (m, 4H, H-3′, H-6′), 1.22–1.08 (m, 3H, H-4′, H-5′). ^13^C NMR (75.4 MHz, DMSO) δ: 178.76 (C=S), 146.20 (C-3), 139.47 (C-1), 130.68 (C-4), 126.10 (C-5), 117.27 (C-2, C-6), 56.34 (C-7′), 40.97 (C-2′, C-6′), 34.70 (C-5′), 34.67 (C-1′), 27.29 (C-4′), 25.48 (C-3′). ESI MS: *m*/*z* = 324.1 [M − H]^−^ (100%). Rf for TLC (CHCl_3_:MeOH (1%)): 0.09.

Crystal data: crystal system triclinic, space group P-1, unit cell dimensions at 120 K: a = 7.273(2) Å, b = 9.652(2) Å, c = 11.862(3) Å, α = 105.57(2)°, β = 105.66(3)°, γ = 96.76(2)°, V = 756.0(3) Å^3^, Z = 2, D_calc_ = 1.431 g/cm^3^, μ = 3.600 mm^−1^, F(000) = 340, θ range 4.08 to 73.77°, reflections collected/independent/observed 10755/2994/2701; max. and min. transmission 1 and 0.75929; Goodness-of-fit on F^2^ 1.084, final *R* indices [I > 2σ(I)] R1 = 0.0395, wR2 = 0.0943; *R* indices (all data) R1 = 0.0456, wR2 = 0.0975, residual electron density max/min 0.45 and−0.54 e Å^−3^. The cycloalkyl group, viz. 1-bicyclo[2.2.1]hept-2-yl, is disordered over two positions with the site occupancy factors being 0.64:0.36. CCDC No. 1849699.

1-(4-chloro-3-nitrophenyl)-3-(2-methylprop-2-en-1-yl)thiourea (**27**)

Yield 29%, light brown powder, m.p. 96–98 °C. FT-IR (KBr, cm^−1^): 3336.7, 3198.9 (ν N-H); 3075.6, 3022.8 (ν arC-H); 1652.3 (ν C=C); 1577.4 (δ N-H); 1535.1 (ν N-O); 1470.4 (ν arC-C, δ alC-H); 1407.5 (δ ar(1,4)C-C); 1347.5 (ν N-O); 1312.2, 1194.6, 1140.4 (δ arC-H); 1040.5 (ν C=S); 882.0 (δ C-Cl); 683.2 (ν C=S). ^1^H NMR (300 MHz, DMSO) δ: 9.99 (br. s, 1H, NH), 8.42 (br. s, 1H, H-2), 8.23 (br. s, 1H, NH), 7.76 (dd, 1H, *J*_1_ = *J*_2_ = 2.4 Hz, H-6), 7.68 (d, 1H, *J* = 9.0 Hz, H-5), 4.84 (s, 2H, H-3′), 4.09 (br. s, 2H, H-1′), 1.72 (s, 3H, H-2′a). ^13^C NMR (75.4 MHz, DMSO) δ: 181.06 (C=S), 147.07 (C-3), 141.78 (C-1), 140.09 (C-5), 131.60 (C-4), 127.49 (C-3′), 118.79 (C-2′), 110.78 (C-2, C-6), 49.19 (C-1′), 20.65 (C-2′a). ESI MS: *m*/*z* = 284.0 [M − H]^−^ (100%). Rf for TLC (CHCl_3_:MeOH (1%)): 0.10.

1-(4-chloro-3-nitrophenyl)-3-prop-2-en-1-ylthiourea (**28**)

Yield 31%, pale yellow crystals, m.p. 112–114 °C. FT-IR (KBr, cm^−1^): 3370.1, 3173.8 (ν N-H); 3072.7, 3002.6 (ν arC-H, ν =C-H); 1641.6 (ν C=C); 1600.7 (δ N-H); 1534.5 (ν N-O); 1471.6 (ν arC-C); 1431.1 (δ =C-H); 1409.4 (δ ar(1,4)C-C); 1349.5 (δ alC-H, δ =C-H); 1311.5 (ν N-O); 1195.9, 1127.9 (δ arC-H); 1041.4 (ν C=S); 839.4 (δ C-Cl); 711.5 (ν C=S). ^1^H NMR (300 MHz, DMSO) δ: 9.97 (s, 1H, NH), 8.40 (d, 1H, *J* = 2.1 Hz, H-2), 8.24 (s, 1H, NH), 7.76 (dd, 1H, *J*_1_ = 2.4 Hz, *J*_2_ = 2.7 Hz, H-6), 7.68 (d, 1H, *J* = 9.0 Hz, H-5), 5.96–5.83 (m, 1H, H-2′), 5.25–5.11 (m, 2H, H-3′), 4.15 (m, 2H, H-1′). ^13^C NMR (75.4 MHz, DMSO) δ: 180.52 (C=S), 146.82 (C-3), 139.78 (C-1), 134.19 (C-5), 131.32 (C-4), 127.31 (C-3′), 118.64 (C-2′), 116.08 (C-2, C6), 46.02 (C-1′). ESI MS: *m*/*z* = 270.0 [M − H]^−^ (100%). Rf for TLC (CHCl_3_:MeOH (1%)): 0.11.

1-(4-chloro-3-nitrophenyl)-3-[3-(methylsulfanyl)propyl]thiourea (**29**)

Yield 35%, light brown powder, m.p. 102–103.5 °C. FT-IR (KBr, cm^−1^): 3330.7, 3171.5 (ν N-H); 3070.3, 2999.3 (ν arC-H); 2941.3, 2915.6 (ν alC-H); 1572.8 (δ N-H); 1531.5 (ν N-O); 1475.5 (ν arC-C); 1455.1 (δ alC-H); 1410.5 (δ ar(1,4)C-C); 1387.7 (δ alC-H); 1351.8 (ν N-O); 1298.9, 1154.7, 1134.1 (δ arC-H); 1043.6 (ν C=S); 887.3 (δ C-Cl); 701.7 (ν C=S). ^1^H NMR (300 MHz, DMSO) δ: 9.88 (br. s, 1H, NH), 8.37 (d, 1H, *J* = 2.1 Hz, H-2), 8.17 (br. s, 1H, NH), 7.73 (dd, 1H, *J*_1_ = *J*_2_ = 1.8 Hz, H-6), 7.67 (d, 1H, *J* = 8.7 Hz, H-5), 3.56–3.54 (m, 2H, H-1′), 2.51–2.49 (m, 2H, H-3′), 2.06–2.05 (m, 3H, H-4′), 1.87–1.78 (m, 2H, H-2′). ^13^C NMR (75.4 MHz, DMSO) δ: 179.85 (C=S), 146.25 (C-3), 139.24 (C-1), 130.78 (C-4), 126.57 (C-5), 117.86 (C-2, C-6), 42.28 (C-1′), 30.04 (C-2′), 27.03 (C-3′), 14.04 (C-4′). ESI MS: *m*/*z* = 318.0 [M − H]^−^ (100%). Rf for TLC (CHCl_3_:MeOH (1%)): 0.09.

1-(4-chloro-3-nitrophenyl)-3-(furan-2-ylmethyl)thiourea (**30**)

Yield 58%, brown powder, m.p. 144–146 °C. FT-IR (KBr, cm^−1^): 3299.1, 3180.4 (ν N-H); 3009.7 (ν arC-H); 1578.2 (δ N-H); 1537.3 (ν N-O); 1478.9 (ν arC-C); 1448.8 (δ alC-H); 1407.9 (δ ar(1,4)C-C); 1364.3 (δ alC-H); 1339.2 (ν N-O); 1297.7, 1190.4, 1139.0 (δ arC-H); 1018.2 (ν C=S); 884.6 (δ C-Cl); 714.8 (ν C=S). ^1^H NMR (300 MHz, DMSO) δ: 9.98 (s, 1H, NH), 8.49 (s, 1H, NH), 8.41 (d, 1H, *J* = 2.1 Hz, H-2), 7.76 (dd, 1H, *J*_1_ = *J*_2_ = 2.4 Hz, H-6), 7.68 (d, 1H, *J* = 8.7 Hz, H-5), 7.62–7.61 (m, 1H, H-5′), 6.42 (dd, 1H, *J*_1_ = 2.1 Hz, *J*_2_ = 1.8 Hz, H-4′), 6.35 (d, 1H, *J*_1_ = 3.3 Hz, H-3′), 4.73 (d, 2H, *J* = 5.1 Hz, H-1′). ^13^C NMR (75.4 MHz, DMSO) δ: 180.27 (C=S), 150.75 (C-2′), 146.55 (C-3), 142.09 (C-5′), 139.42 (C-1), 131.07 (C-4), 127.09 (C-5), 118.62 (C-2), 118.37 (C-6), 110.25 (C-4′), 107.44 (C-3′). ESI MS: *m*/*z* = 310.0 [M − H]^−^ (100%). Rf for TLC (CHCl_3_:MeOH (1%)):0.15.

The NMR spectra of compounds **20**–**24**, **27**–**30** can be found in Supplementary Materials.

### 3.2. X-ray Crystalography

The X-ray diffraction intensities were measured for **20** and **24** on a SuperNova diffractometer with monochromatized CuKα radiation (*λ* = 1.54184 Å). Data sets were collected using the *ω* scan technique. The programs CrysAlisPro [46] were used for data collection, cell refinement and data reduction; empirical absorption correction was applied using spherical harmonics implemented in SCALE3 ABSPACK scaling algorithm. The structure was solved by direct methods using SHELXS-97 and refined by the full-matrix least-squares on *F*^2^ using the SHELXL-97 [47]. Non-hydrogen atoms were refined with anisotropic displacement parameters. Hydrogen atoms were positioned geometrically and allowed to ride on their parent atoms, with *U_iso_*(H) = 1.2 *U_eq_*(C). 

### 3.3. In Vitro Evaluation of Antimicrobial Activity

The antimicrobial activity of the compounds was tested on Gram-positive bacteria (*Staphylococcus aureus* NCTC 4163, *Staphylococcus aureus* ATCC 25923, *Staphylococcus aureus* ATCC 6538, *Staphylococcus aureus* ATCC 29213, *Staphylococcus epidermidis* ATCC 12228, *Staphylococcus epidermidis* ATCC 35984, *Enterococcus faecalis* ATCC 29212, *Bacillus subtilis* ATCC 6633, *Bacillus cereus* ATCC 11778, *Micrococcus luteus* ATCC 9341, *Micrococcus luteus* ATCC 10240); Gram-negative rods (*Escherichia coli* ATCC 10538, *Escherichia coli* ATCC 25922, *Escherichia coli* NCTC 8196, *Proteus vulgaris* NCTC 4635, *Pseudomonas aeruginosa* ATCC 15442, *Pseudomonas aeruginosa* NCTC 6749, *Pseudomonas aeruginosa* ATCC 27863, *Bordetella bronchiseptica* ATCC 4617) and yeasts (*Candida albicans* ATCC 10231, *Candida albicans* ATCC 90028, *Candida parapsilosis* ATCC 22019). Other microorganisms used were obtained from the collection of the Department of Pharmaceutical Microbiology, Medical University of Warsaw, Poland.

Antibacterial activity was examined by the disc-diffusion method under standard conditions using Mueller-Hinton II agar medium (Becton Dickinson) according to CLSI (previously NCCLS) guidelines [39]. Antifungal activities were assessed using Mueller-Hinton agar + 2% glucose and 0.5 µg/mL Methylene Blue Dye Medium [40]. Sterile filter paper discs (9 mm diameter, Whatman No 3 chromatography paper) were dripped with tested compound solutions (in DMSO) to load 400 μg of a given compound per disc. Dry discs were placed on the surface of appropriate agar medium. The results (diameter of the growth inhibition zone) were read after 18 h of incubation at 35 °C. Minimal Inhibitory Concentration (MIC) was tested by the twofold serial microdilution method (in 96-well microtiter plates) using Mueller-Hinton Broth medium (Beckton Dickinson) for bacteria or RPMI-1640 medium for *Candida* species according to CLSI guidelines [41,42]. The stock solution of tested agent was prepared in DMSO and diluted in sterile water. Concentrations of tested agents ranged from 0.125 to 512 µg/mL. The final inoculum of all studied microorganisms was 10^5^ CFU/ mL^−1^ (colony forming units per mL). Minimal inhibitory concentrations (the lowest concentration of a tested agent that prevents visible growth of a microorganism) were read after 18 h (bacteria) or 24 h (yeasts) of incubation at 35 °C. 

### 3.4. Cytotoxicity and Anti-HIV Assays

Cell line supporting the multiplication of Human Immunodeficiency Virus type-1 (HIV-1) was the CD4+ human T-cells, derived from human hematological tumors, containing an integrated HTLV-1 genome (MT-4). Cells were purchased from American Type Culture Collection (ATCC). Human Immunodeficiency Virus type-1 (HIV-1) IIIB laboratory strain was obtained from the supernatant of the persistently infected H9/IIIB cells (NIH 1983).

Exponentially growing MT-4 cells were seeded at an initial density of 1 × 10^5^ cells/mL in 96-well plates in RPMI-1640 medium, supplemented with 10% fetal bovine serum (FBS), 100 units/mL penicillin G and 100 μg/mL streptomycin. Cell cultures were then incubated at 37 °C in a humidified, 5% CO_2_ atmosphere, in the absence or presence of serial dilutions of tested compounds. Cell viability was determined after 96 h at 37 °C by the 3-(4,5-dimethylthiazol-2-yl)-2,5-diphenyl-tetrazolium bromide (MTT) method.

The activity of compounds against HIV-1 was based on inhibition of virus-induced cytopathogenicity in MT-4 cell acutely infected with a multiplicity of infection (m.o.i.) of 0.01. Briefly, 50 μL of RPMI containing 1 × 10^4^ MT-4 cells were added to each well of flat-bottom microtiter trays, containing 50 μL of RPMI without or with serial dilutions of test compounds. Then, 20 μL of a HIV-1 suspension containing 100 CCID_50_ were added. After a 4-day incubation at 37 °C, cell viability was determined by the MTT method.

### 3.5. Antiproliferative Assays

Cell lines derived from human hematological tumors were: CD4+ human T-cells containing an integrated HTLV-1 genome (MT-4); CD4+ human acute T-lymphoblastic leukemia (CCRF-CEM); human splenic B-lymphoblastoid cells (WIL-2NS); human acute B-lymphoblastic leukemia (CCRF-SB). Cells were seeded at an initial density of 1 × 10^5^ cells/mL in 96 well plates in RPMI-1640 medium supplemented with 10% fetal calf serum (FCS), 100 units/mL penicillin G and 100 μg/mL streptomycin.

Cell lines derived from human solid tumors were: Skin melanoma (SK-MEL-28); prostate carcinoma (DU-145). Normal tissues foreskin fibroblasts (CRL-7065) were also used. Cells were seeded at 1 × 10^5^ cells/mL in 96 well plates in specific media supplemented with 10% FCS and antibiotics, as above. Cell cultures were then incubated at 37 °C in a humidified, 5% CO_2_ atmosphere in the absence or presence of serial dilutions of test compounds. Cell viability was determined after 96 h at 37 °C by the MTT method.

### 3.6. Cytotoxic Activity in HaCaT Cells

#### 3.6.1. Cell Culture: Conditions and Treatments

Human immortal keratinocyte cell line from adult human skin (HaCaT) was purchased from American Type Culture Collection (Rockville, MD, USA), and cultured in Dulbecco’s Modified Eagle’s Medium (DMEM), supplemented with antibiotics (penicillin and streptomycin), 10% heat-inactivated FBS-fetal bovine serum (Gibco Life Technologies, Carlsbad, CA, USA) at 37 °C and 5% CO_2_ atmosphere. Cells were passaged using trypsin-EDTA (Gibco Life Technologies, Carlsbad, CA, USA) and cultured in 24-well plates (2.5 × 10^4^ cells per well). Experiments were conducted in DMEM with 2% FBS. 

#### 3.6.2. Cell Viability Assessment (Mitochondrial Function Assessment)

The cell viability assay was assessed by the determination of MTT salt (3-(4,5-dimethylthiazol-2-yl)-2,5-diphenyltetrazolium bromide, Sigma-Aldrich Chemie, Buchs, Germany) conversion by mitochondrial dehydrogenase. The cells were incubated for 24 h in 24-well plates with tested compounds (**11**, **13** and **25**) at given concentrations, and subsequently for another 2 h with 0.5 mg/mL of MTT solution, which was converted in living cells under the effect of mitochondrial dehydrogenase into insoluble formazan. Then the converted dye was solubilized with the use of 0.04 M HCl in absolute isopropanol. Absorbance of solubilized formazan was measured spectrophotometrically at 570 nm (using Epoch microplate reader, BioTek Inc., Winooski, VT, USA, equipped with Gen5 software (BioTech Instruments, Inc., Biokom, Janki, Polska).

Cell viability was presented as a percent of MTT in the treated cells versus the control (cells incubated in serum-free DMEM without extracts). The relative MTT level (%) was calculated as [A]/[B] × 100, where [A] is the absorbance of the test sample and [B] is the absorbance of the control sample containing the untreated cells. The decreased relative MTT level indicates decreased cell viability.

#### 3.6.3. Lactate Dehydrogenase Release Assay (Cellular Membrane Integrity Assessment)

Release of lactate dehydrogenase (LDH) from the cytosol to culture medium is a marker of cell death. The assay was performed after 24 h incubation of HaCaT cells in 24-well plates with investigated concentrations of each compound. The activity of LDH released from the cytosol of damaged cells to the supernatant was measured using the protocol of the cytotoxicity detection kit LDH test described by the manufacturer (Roche Diagnostics, Berlin, Germany). Absorbance was measured at 490 nm using a microplate reader (using Epoch microplate reader, BioTek Inc., Winooski, VT, USA) equipped with Gen5 software (BioTech Instruments, Inc., Biokom, Janki, Polska)

Compounds mediated cytotoxicity expressed as the LDH release (%) was determined by the following equation: [(A test sample—A low control)/(A high control—A low control)] × 100% (A-absorbance); where “low control” were cells in DMEM with 2% FBS without tested compounds, and “high control” were cells incubated in DMEM with 2% FBS with 10% Triton X-100 (100% LDH release).

#### 3.6.4. Proliferation Rate in Cultures of HaCaT Cells

After 48 h incubation with the lowest concentration of the tested compound the increased number of viable cells were assessed by direct counting of cells using trypan blue with a Countess Automated Cell Counter (Invitrogen, Carlsbad, CA, USA). The results are presented as a % of viable and dead cells versus control.

## 4. Conclusions

To sum up, this work has revealed the synthesis of new thiourea derivatives and their in vitro antibacterial activity against clinically relevant pathogens. The title compounds showed potent to moderate antimicrobial activity. Representative aryl-thioureas **20**, **22**, **23** and bicyclic-thiourea derivative **24** were even more active towards selected clinical staphylococci than the reference drug, Ciprofloxacin. To complete the biological profile of the presented thiourea series, their cytotoxic activities in MT-4 cells, as well as human hematological and solid tumors cell lines were assessed. Several compounds (**1**, **4**, **6**, **9**–**11**, **13**, **14**, **17**, **19**) turned out cytotoxic in the low micromolar range (CC_50_ < 10 µM). Performed studies proved that synthesized derivatives weakly to moderately influenced the viability and growth of the human immortal keratinocyte cell line (HaCaT).

## Figures and Tables

**Table 1:** Cytotoxicity and anti-HIV activity of 4-chloro-3-nitrophenylthiourea derivatives **1**–**30**.

Comp.	R	MT-4 * ^1^ CC_50_ [µM]	HIV-1_IIIB_ * ^2^ EC_50_ [µM]
**1**	2-chlorophenyl	8.8	>8.8
**2**	3-chlorophenyl	7.0	>7.0
**3**	4-chlorophenyl	7.0	>7.0
**4**	2-bromophenyl	9.6	>9.7
**5**	3-bromophenyl	8.0	>8.0
**6**	4-bromophenyl	6.6	>6.6
**7**	2-fluorophenyl	13.0	>13.0
**8**	3-fluorophenyl	9.0	>9.0
**9**	4-fluorophenyl	7.0	>7.0
**10**	3-chloro-4-fluorophenyl	7.3	>7.3
**11**	3,4-dichlorophenyl	7.9	>7.9
**12**	2,4-dichlorophenyl	8.0	>8.0
**13**	3-chloro-4-methylphenyl	8.0	>8.0
**14**	5-chloro-2-methylphenyl	8.2	>8.2
**15**	3-(trifluoromethyl)phenyl	8.0	>8.0
**16**	4-(trifluoromethyl)phenyl	7.0	>7.0
**17**	4-iodophenyl	7.8	>7.8
**18**	4-nitrophenyl	7.0	>7.0
**19**	4-cyanophenyl	8.2	>8.2
**20**	4-methylphenyl	14.6	>14.6
**21**	4-methoxyphenyl	35.0	>35.0
**22**	4-butyl-2-methylphenyl	18.7	>18.7
**23**	phenyl	23.0	>23.0
**24**	bicyclo[2.2.1]hept-2-yl	16.0	>16.0
**25**	benzyl	36.7	>36.7
**26**	1-phenylethyl	16.7	>16.7
**27**	(2-methyl)prop-2-en-1-yl	15.5	>15.5
**28**	prop-2-en-1-yl	43.0	43.0
**29**	3-(methylsulfanyl)propyl	33.7	>33.7
**30**	furan-2-ylmethyl	25.6	>25.6

^1^ Compound concentration (µM) required to reduce the viability of mock-infected MT-4 cells by 50%, as determined by the MTT method; ^2^ Compound concentration (µM) was required to achieve 50% protection of MT-4 cells from the HIV-1 induced cytopathogenicy, as determined by the MTT method; * Reference compound—Efavirenz (CC_50_ = 46 µM, EC_50_ = 0.002 µM).

**Table 2:** Activity of compounds against standard bacterial strains, expressed as minimal inhibitory concentrations (MIC, µg/mL).

Comp.	*S. aureus* NCTC 4163	*S. aureus* ATCC 25923	*S. aureus* ATCC 6538	*S. aureus* ATCC 29213	*S. epidermidis* ATCC 35984	*S. epidermidis* ATCC 12228	*B. subtilis* ATCC 6633	*B. cereus* ATCC 11778	*E. faecalis* ATCC 29212	*M. luteus* ATCC 9341	*M. luteus* ATCC 10240
**20**	2	2	4	2	4	4	4	4	‒	4	4
**22**	4	2	2	2	4	4	4	4	‒	4	2
**23**	16	16	16	16	16	16	8	8	16	4	8
**24**	8	8	8	8	16	16	8	8	‒	32	16
**27**	16	16	16	16	16	16	16	8	64	16	32
**28**	64	32	32	64	32	32	32	32	64	32	32
**29**	16	16	16	16	16	16	16	8	64	16	32
**30**	16	8	16	16	32	32	16	16	32	16	16
* Ref.	0.25	0.5	0.25	0.5	0.25	0.25	<0.12	0.25	1	1	2

* Ref.—Ciprofloxacin.

**Table 3:** Activity of compounds against hospital methicillin-resistant strains of *Staphylococcus epidermidis* (MRSE)—minimal inhibitory concentrations (MIC, µg/mL).

Comp.	*S. epidermidis* MRSE *403/11*	*S. epidermidis* MRSE *404/11*	*S. epidermidis* MRSE *405/11*	*S. epidermidis* MRSE *406/11*	*S. epidermidis* MRSE *407/11*	*S. epidermidis* MRSE *409/11*	*S. epidermidis* MRSE *411/11*	*S. epidermidis* MRSE *412/11*	*S. epidermidis* MRSE *413/11*	*S. epidermidis* MRSE *422/11*
**20**	16	16	16	16	16	16	16	32	16	32
**22**	4	8	4	8	4	4	4	8	8	8
**23**	16	16	16	16	16	16	16	32	16	16
**24**	4	4	4	8	4	4	4	4	4	4
**27**	32	32	32	32	32	32	32	64	32	64
**28**	64	64	64	64	64	64	64	128	64	128
**29**	32	32	32	32	32	32	32	32	32	32
**30**	32	32	32	64	32	32	32	32	32	32
* Ref.	16	64	4	0.5	64	2	4	64	32	4

* Ref.—Ciprofloxacin.

**Table 4:** Antiproliferative activity of selected compounds against human leukaemia/lymphoma, solid tumor and normal cell lines.

Comp.	MT-4	CCRF-CEM	WIL-2NS	CCRF-SB	SK-MEL-28	DU145	CRL7065
	^1^ CC_50_						
**1**	8.8	13	14	14	8.7	7.6	18
**4**	9.6	22	30	22	15	8.0	20
**6**	6.6	6.0	7.0	8.0	6.0	6.0	30
**9**	7.0	9.1	11	9.4	20	8.4	22
**10**	7.3	5.0	7.0	8.8	6.6	6.5	16
**11**	7.9	8.0	9.9	8.6	8.5	6.0	16
**13**	8.0	4.0	7.6	6.7	6.0	7.0	16
**14**	8.2	9.4	10	9.8	9.0	7.8	18
**17**	7.8	6.0	7.0	8.3	6.0	11	30
**19**	8.2	9.1	10	9.2	9.0	8.0	20
* Ref	0.004	0.003	0.005	0.004	0.07	0.08	0.3

Data represent mean values for three independent determinations; ^1^ Compound concentration (µM) required to reduce cell proliferation by 50% under conditions allowing untreated controls to undergo at least three consecutive rounds of multiplication, as determined by the MTT method; * Ref.—Camptotecin.

## Supplementary Materials

Supplementary materials can be found online. The experimental details and final atomic parameters for **20** and **24** have been deposited with the Cambridge Crystallographic Data Centre as supplementary material (CCDC ID: 1849698 and 1849698, respectively). Copies of the data can be obtained free of charge on request via www.ccdc.cam.ac.uk/data_request/cif, or by emailing data_request@ccdc.cam.ac.uk, or by contacting The Cambridge Crystallographic Data Centre, 12, Union Road, Cambridge CB2 1EZ, UK; fax: +44 1223 336033.
