# Supplementary material for: Disubstituted 4-Chloro-3-nitrophenylthiourea Derivatives: Antimicrobial and Cytotoxic Studies
Source: Molecules. 2018 Sep 21;23(10):2428. doi: 10.3390/molecules23102428 (PMC6222614; doi:10.3390/molecules23102428)
Supplement: Supplementary file 1 [file molecules-23-02428-s001.pdf]

Supplementary material

Disubstituted 4-chloro-3-nitrophenylthiourea derivatives: antimicrobial and cytotoxic studies

Anna Bielenica [1,*], Giuseppina Sanna [2], Silvia Madeddu [2], Gabriele Giliberti [2], Joanna Stefańska [3], Anna E. Kozioł [4], Oleksandra Savchenko [5], Paulina Strzyga-Łach [1], Alicja Chrzanowska [1], Grażyna Kubiak-Tomaszewska [6], Marta Struga [1,7]

[1] Chair and Department of Biochemistry, Medical University, 02-097 Warszawa, Poland; abielenica@wum.edu.pl (A.B.), paulina.strzyga@gmail.com (P.S.-Ł.), chrzanow.a@gmail.com (A.Ch.)

[2] Department of Biomedical Sciences, Section of Microbiology and Virology, University of Cagliary, 09042 Cittadella Universitaria Monserrato, Italy; ggiliberti@unica.it (G.G.), g.sanna@unica.it (G.S.), silvia.madeddu@unica.it (G.M.)

[3] Department of Pharmaceutical Microbiology, Medical University, 02-007 Warszawa, Poland; jstefanska@wum.edu.pl (J.S.)

[4] Faculty of Chemistry, Maria Curie-Sklodowska University; 20–031 Lublin, Poland; anna.koziol@poczta.umcs.lublin.pl (A.E.K.)

[5] Ecotech Complex, Maria Curie-Sklodowska University; 20–031 Lublin, Poland; oleksandra.savchenko@poczta.umcs.lublin.pl (O.S.)

[6] Department of Biochemistry and Pharmacogenomics, Faculty of Pharmacy, Medical University of Warsaw, 02-097 Warszawa, Poland; grazynakubiak@poczta.onet.pl (G.K.-T)

[7] Laboratory of Centre for Preclinical Research, Medical University of Warsaw, 02-097 Warszawa, Poland; mstruga@wum.edu.pl (M.S.)

^{1}H NMR and ^{13}C NMR spectra of compounds 20-24 and 27-30.

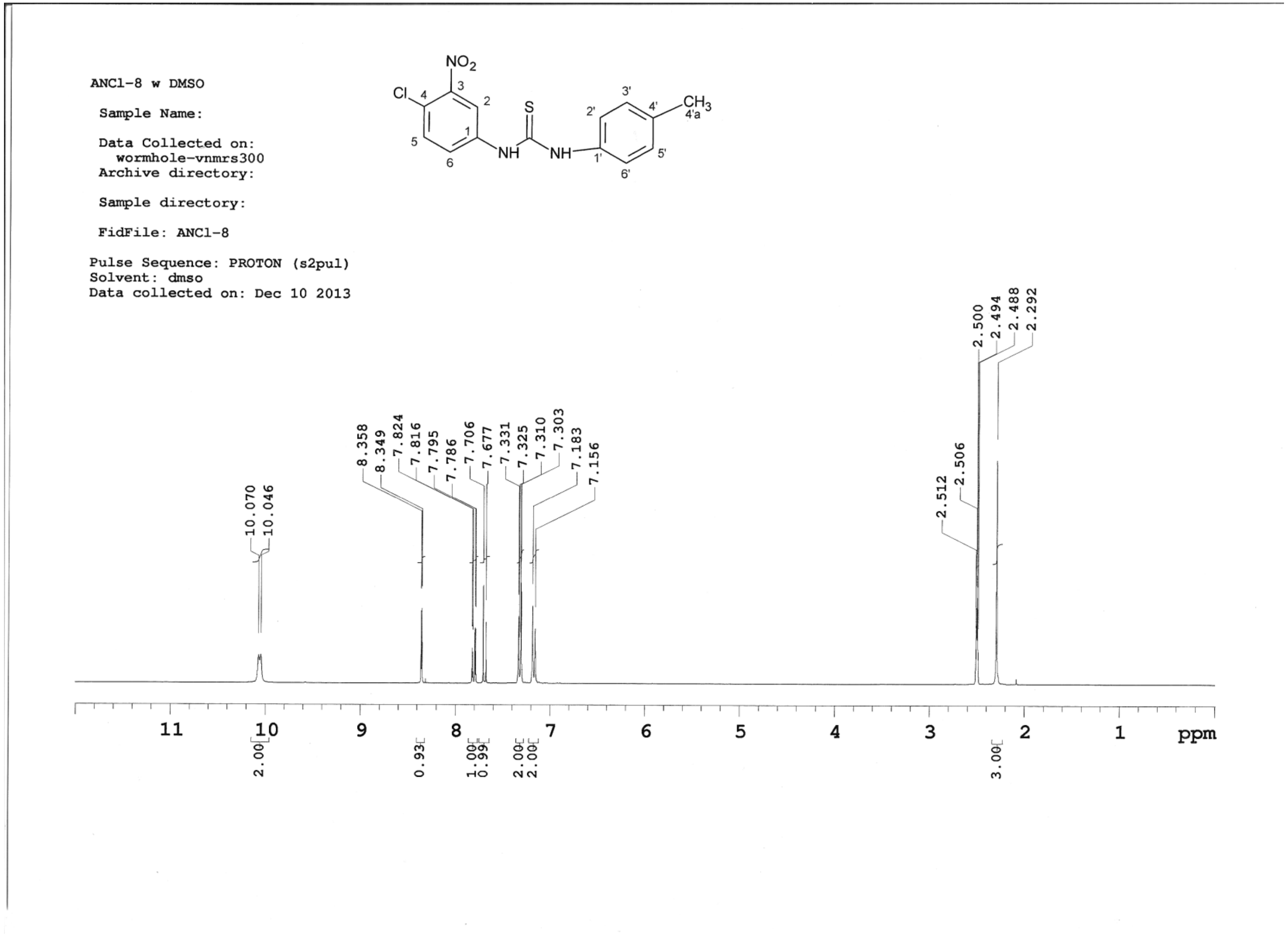

Spectrum 1. ^{1}H NMR of compd **20** (300 MHz, DMSO-d_6).

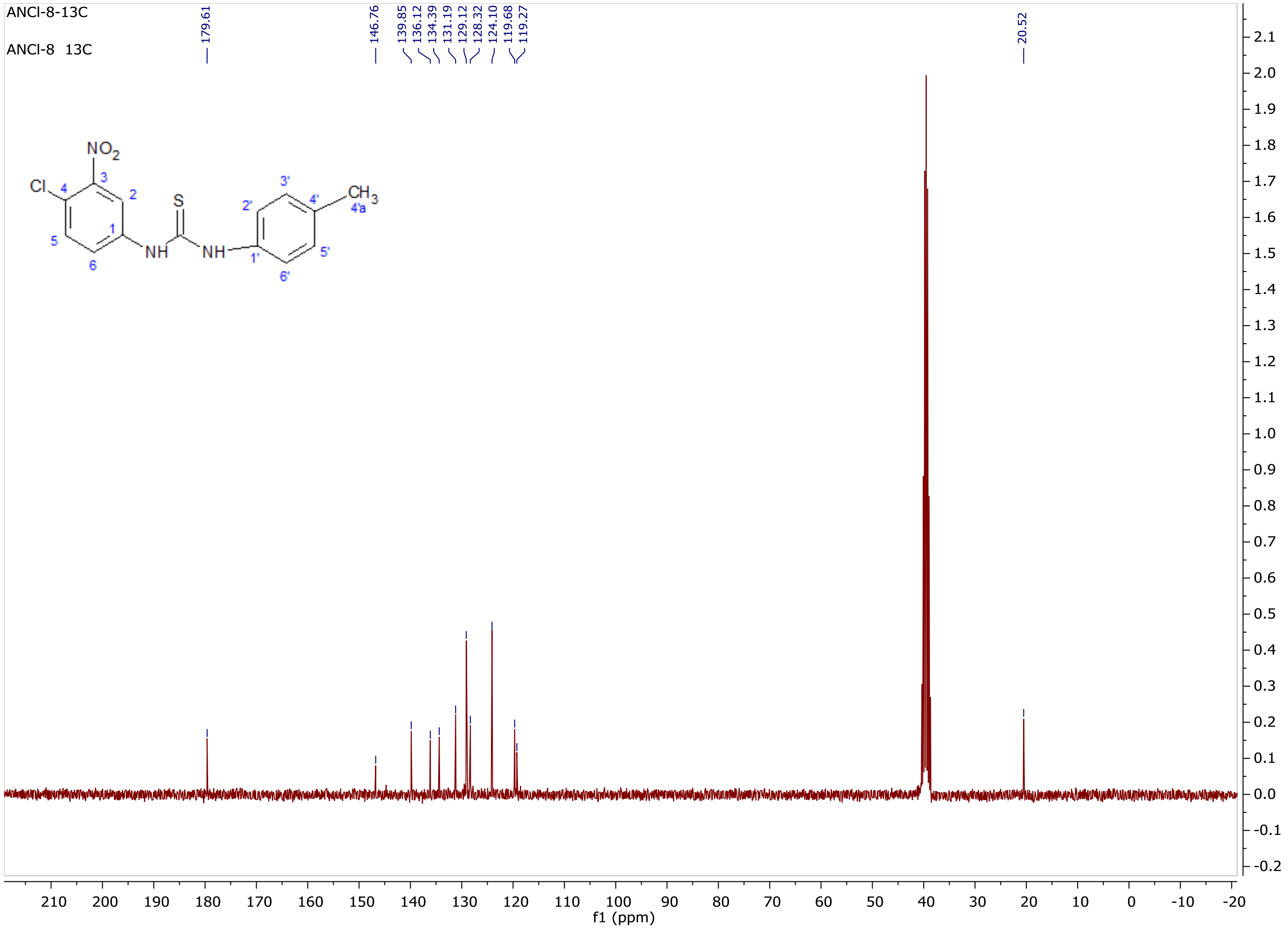

Spectrum 2. ^{13}C NMR of compd **20** (75.4 MHz, DMSO-d$_6$).

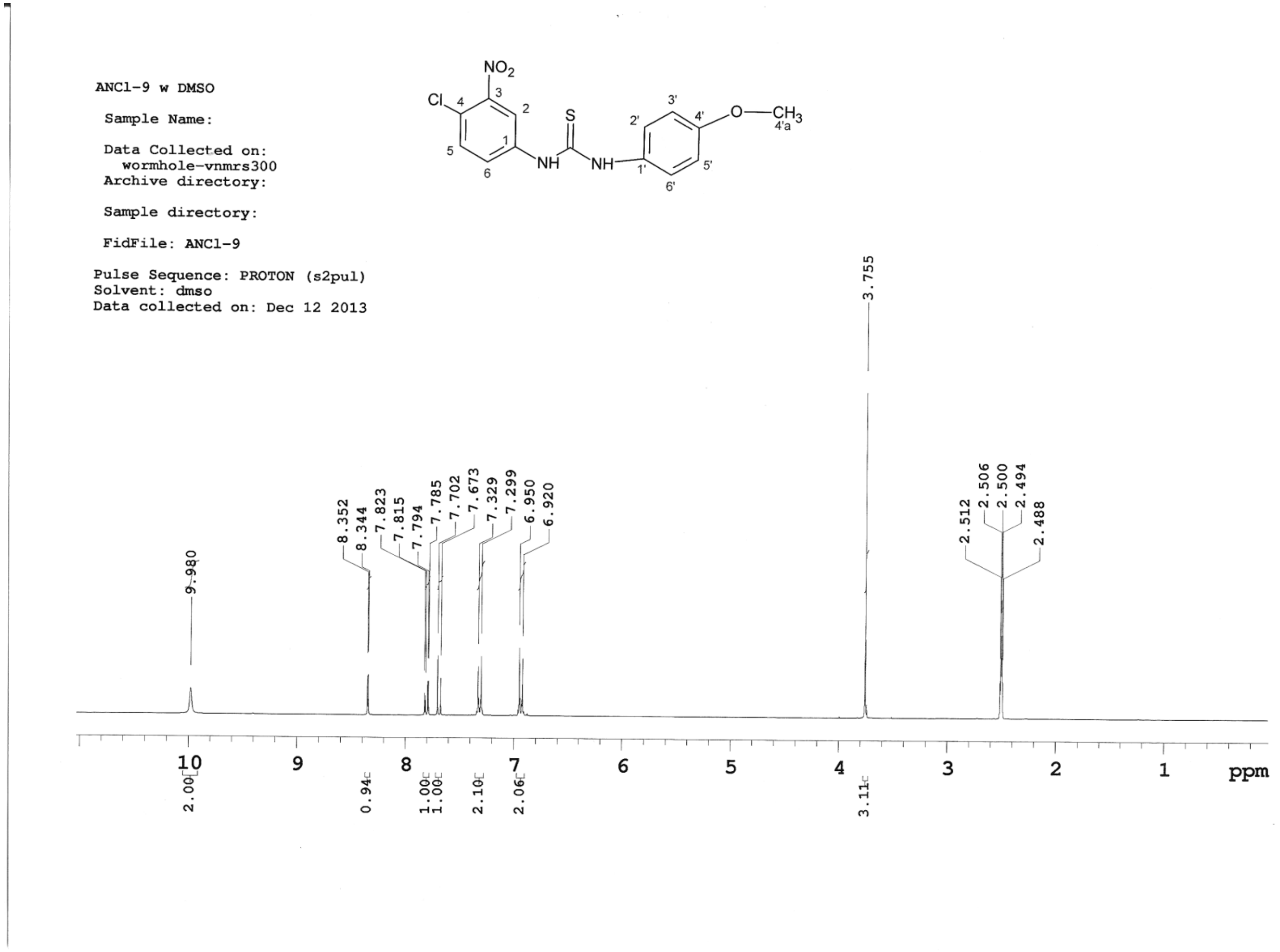

Spectrum 3. ^{1}H NMR of compd **21** (300 MHz, DMSO-d_6).

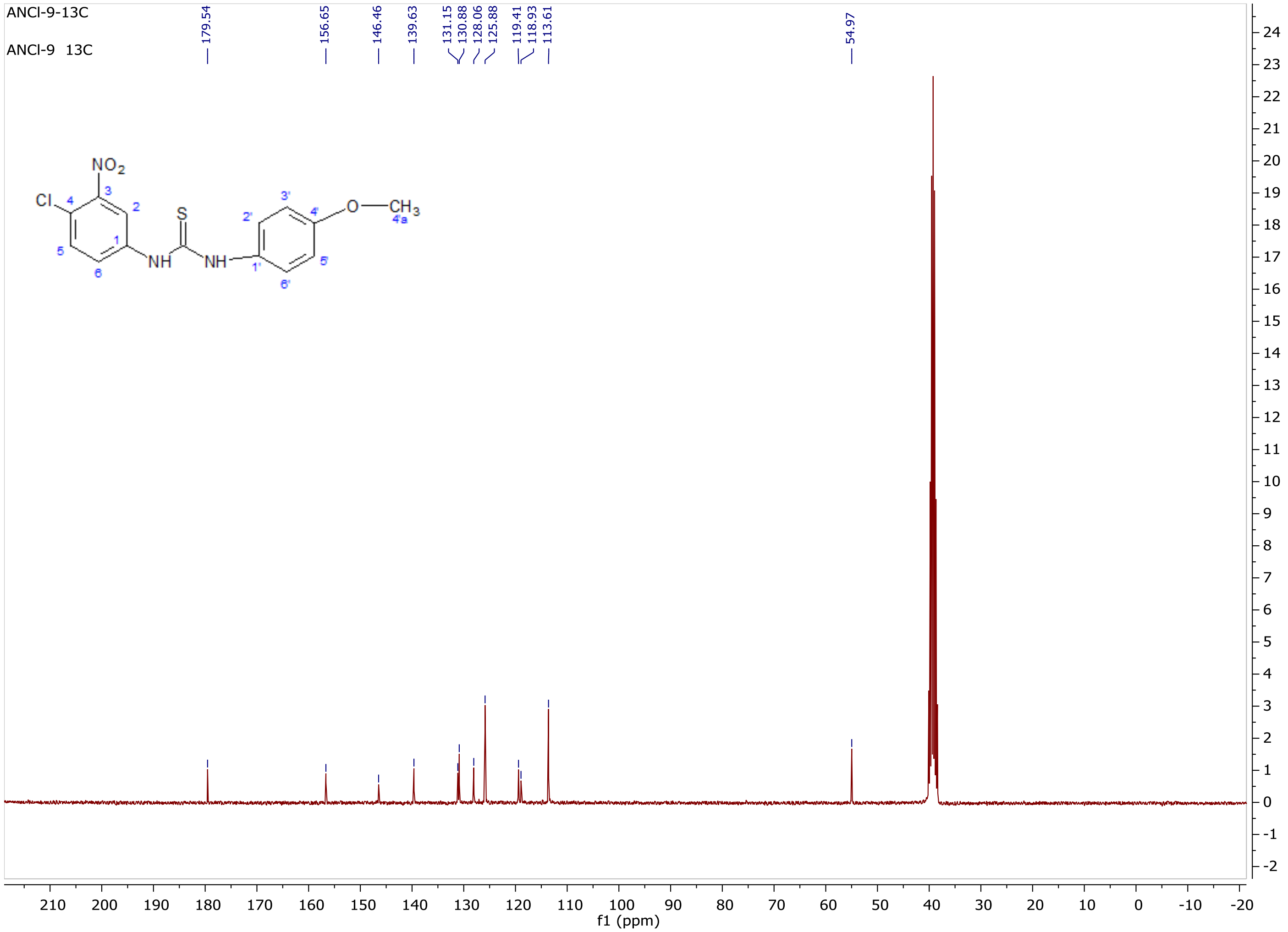

Spectrum 4. ^{13}C NMR of compd **21** (75.4 MHz, DMSO-d_6).

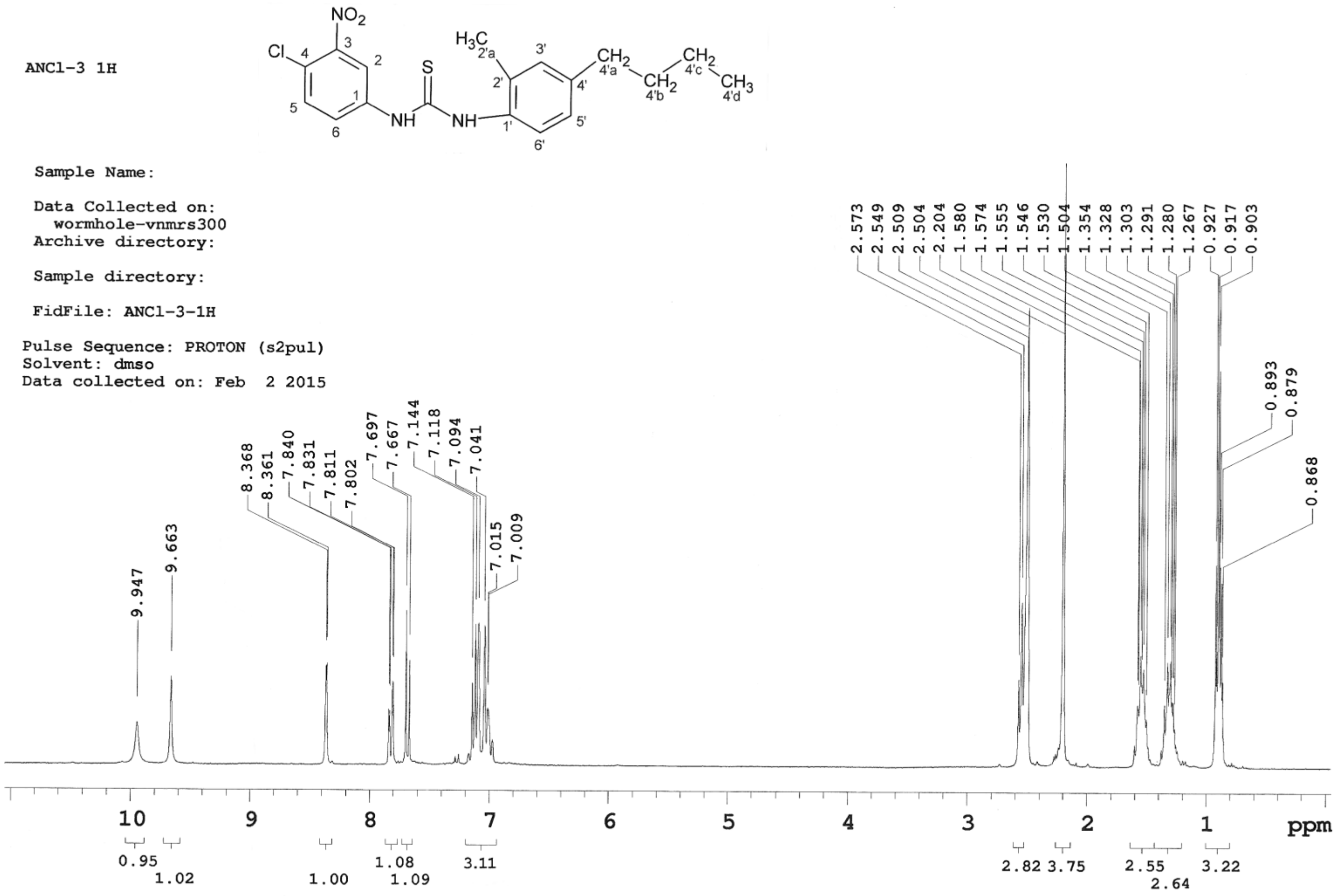

Spectrum 5. ^{1}H NMR of compd **22** (300 MHz, DMSO-d_6).

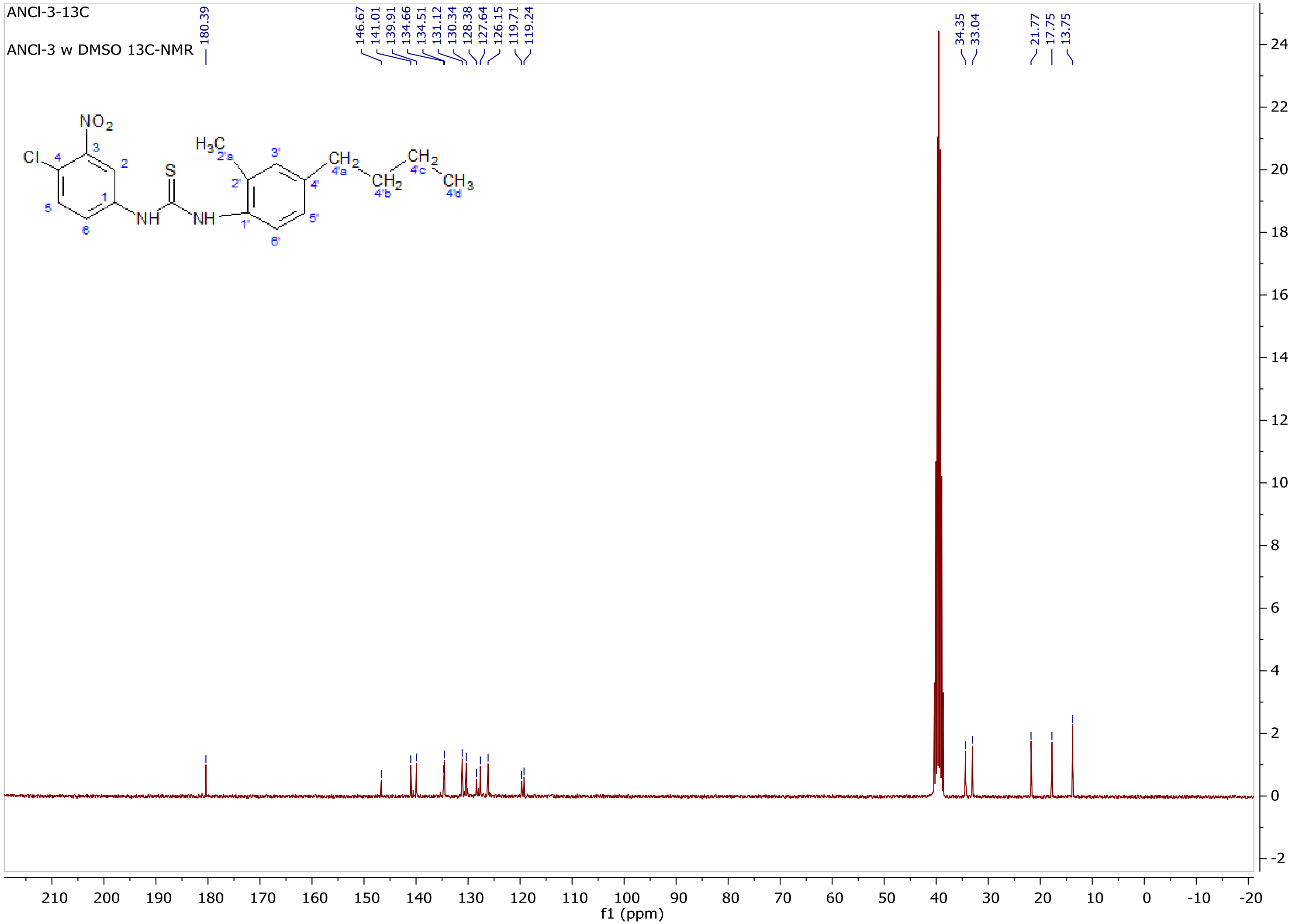

Spectrum 6. ^{13}C NMR of compd **22** (75.4 MHz, DMSO-d_6).

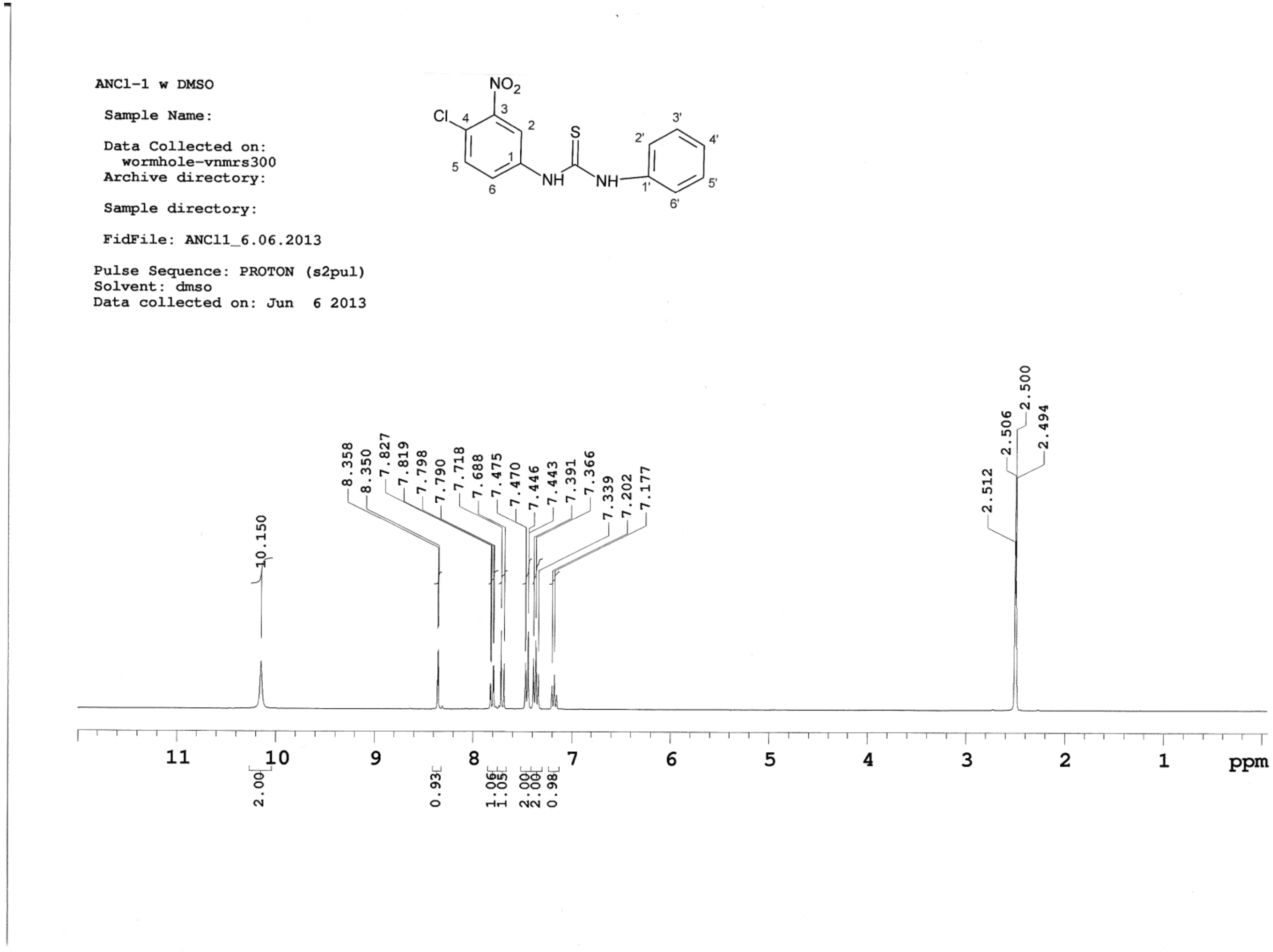

Spectrum 7. ^{1}H NMR of compd **23** (300 MHz, DMSO-d_6).

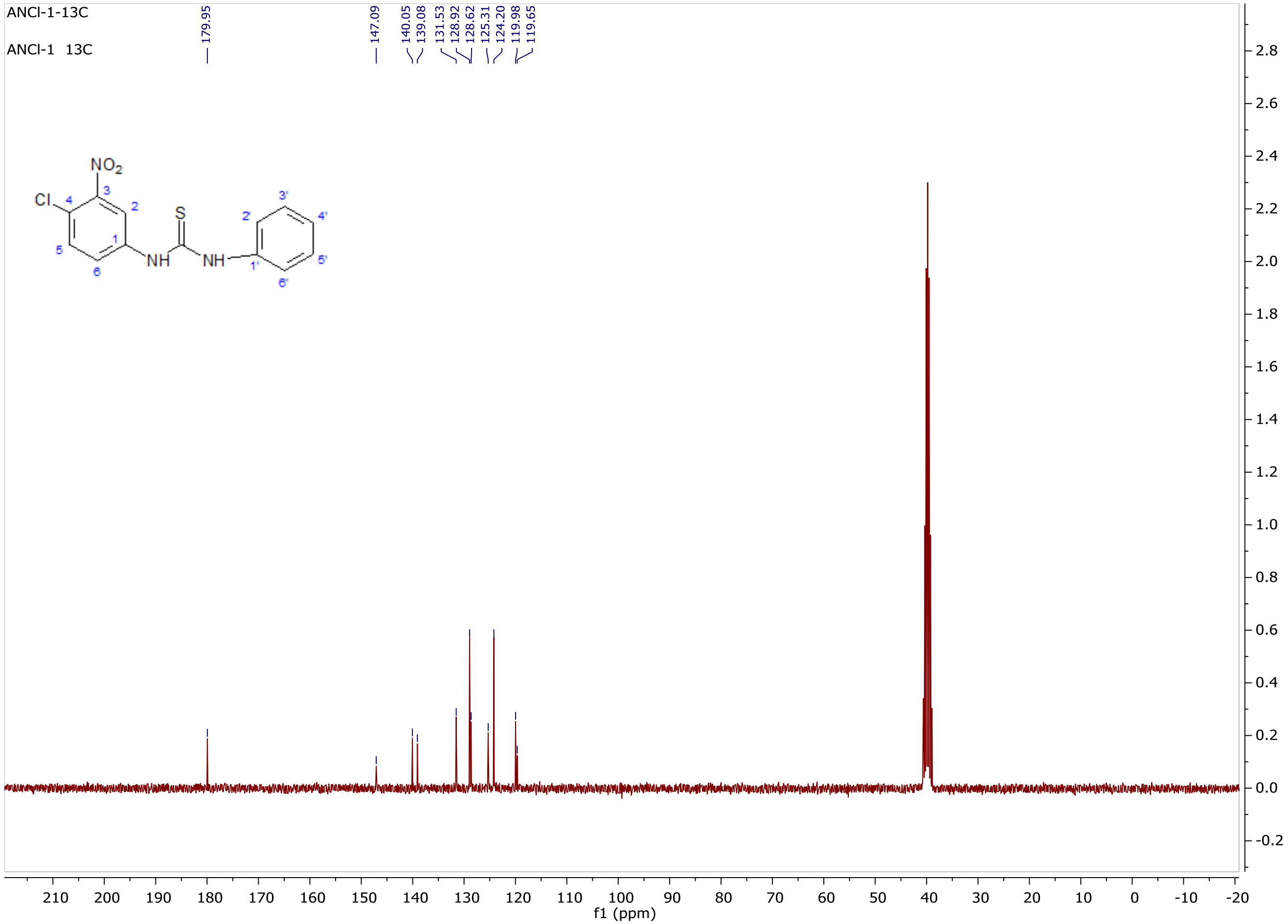

Spectrum 8. ^{13}C NMR of compd **23** (75.4 MHz, DMSO-d$_6$).

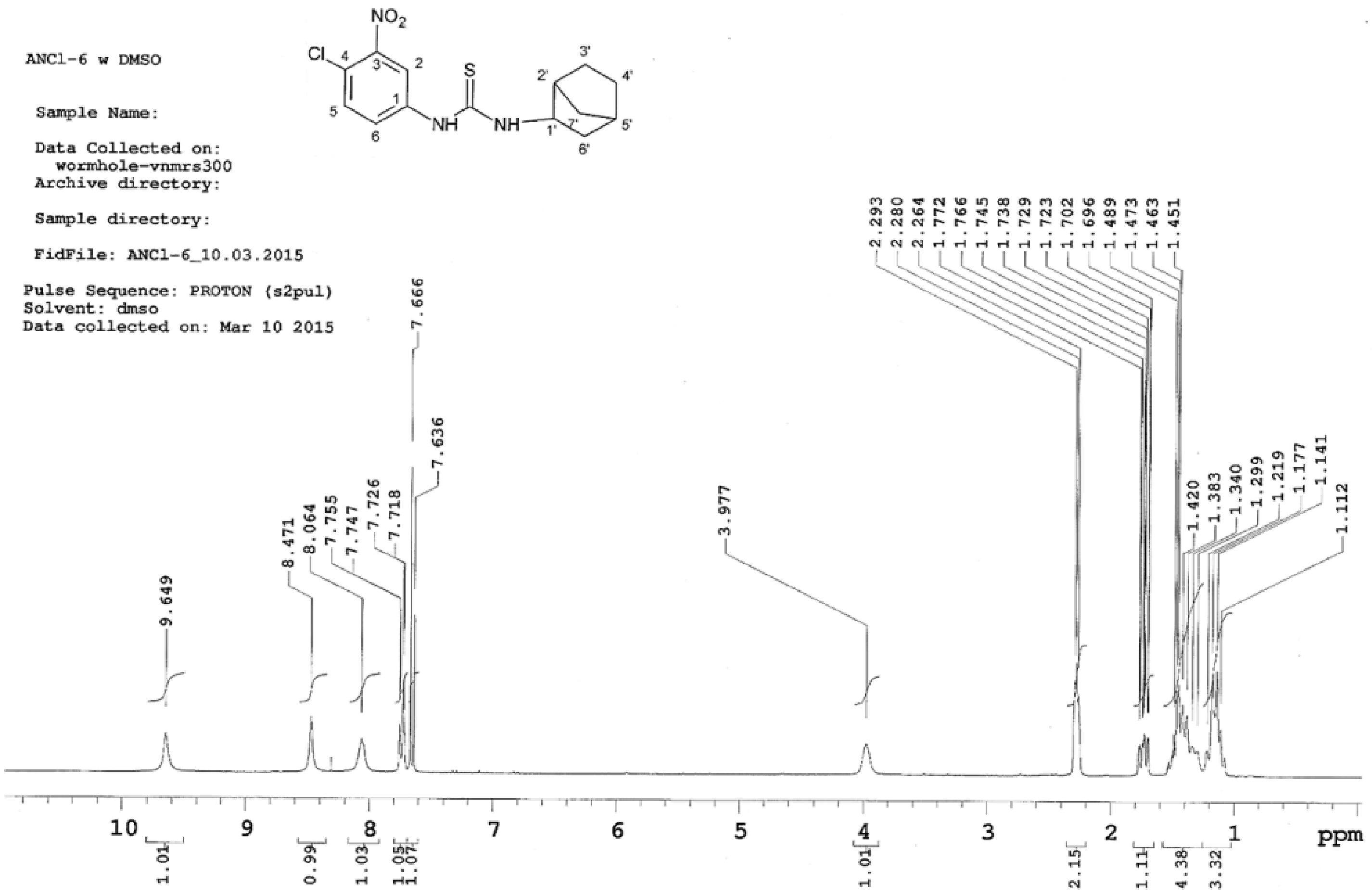

Spectrum 9. ^{1}H NMR of compd **24** (300 MHz, DMSO-d_6).

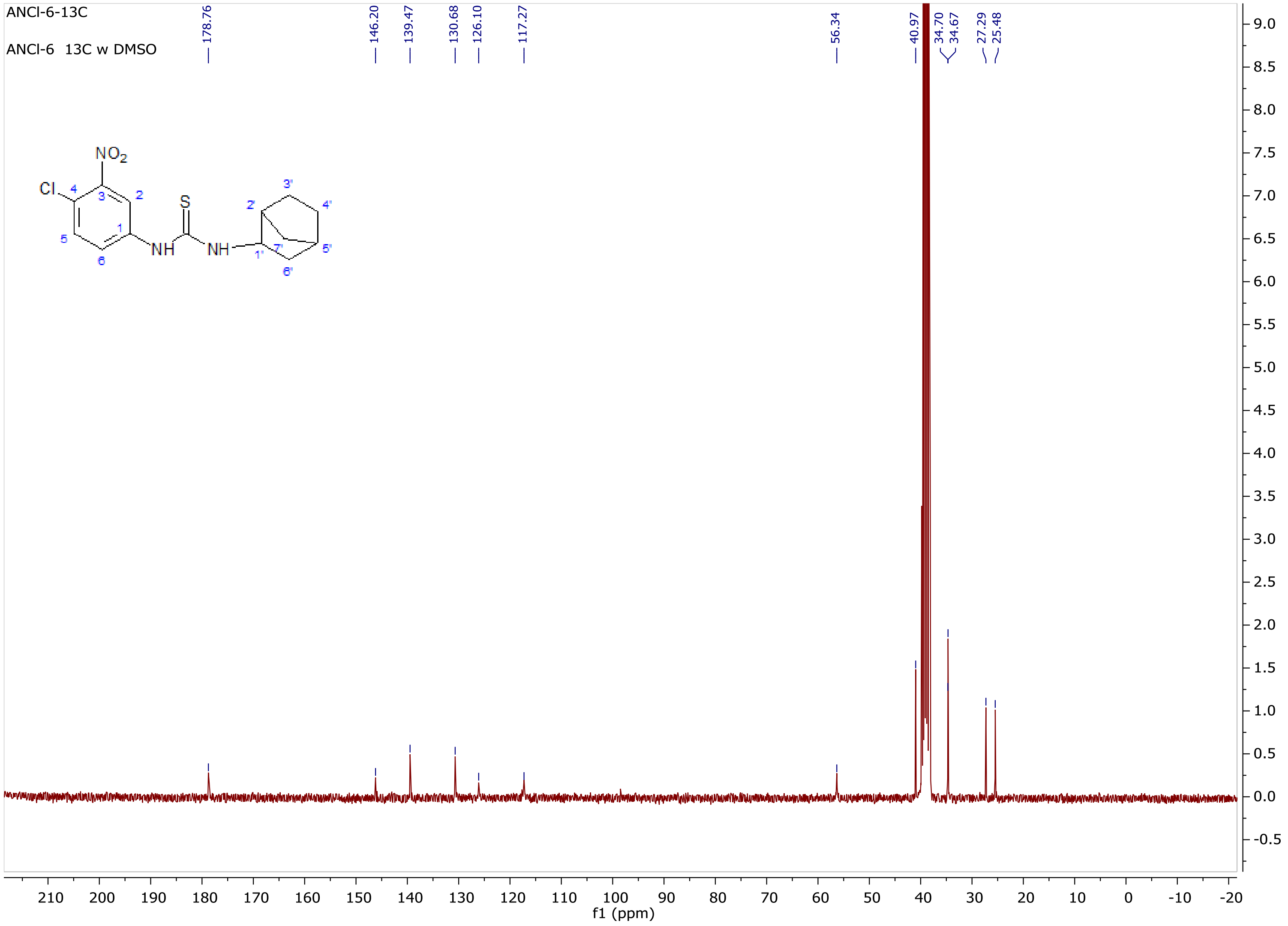

Spectrum 10. ^{13}C NMR of compd **24** (75.4 MHz, DMSO-d_6).

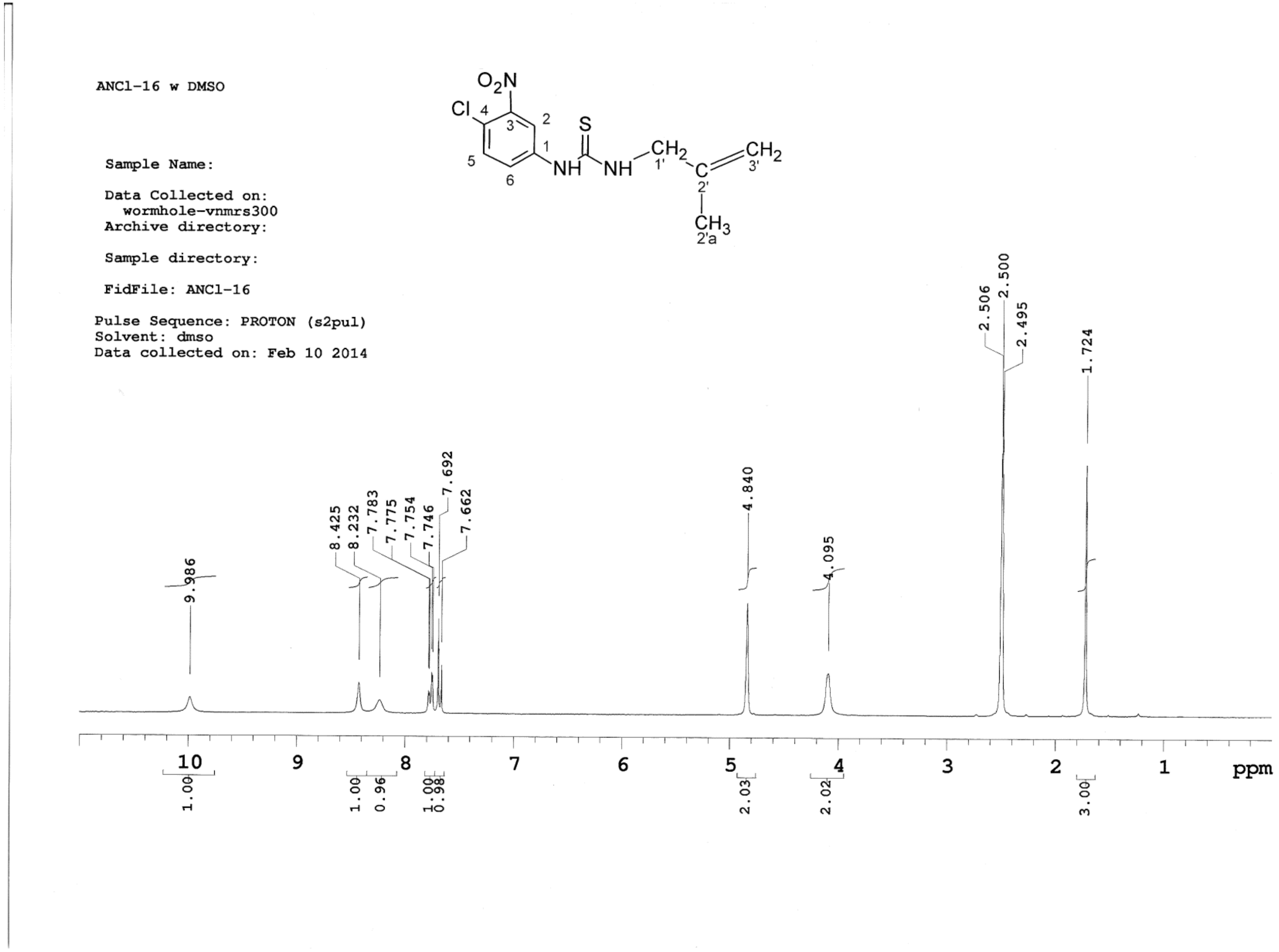

Spectrum 11. ^{1}H NMR of compd **27** (300 MHz, DMSO-d_6).

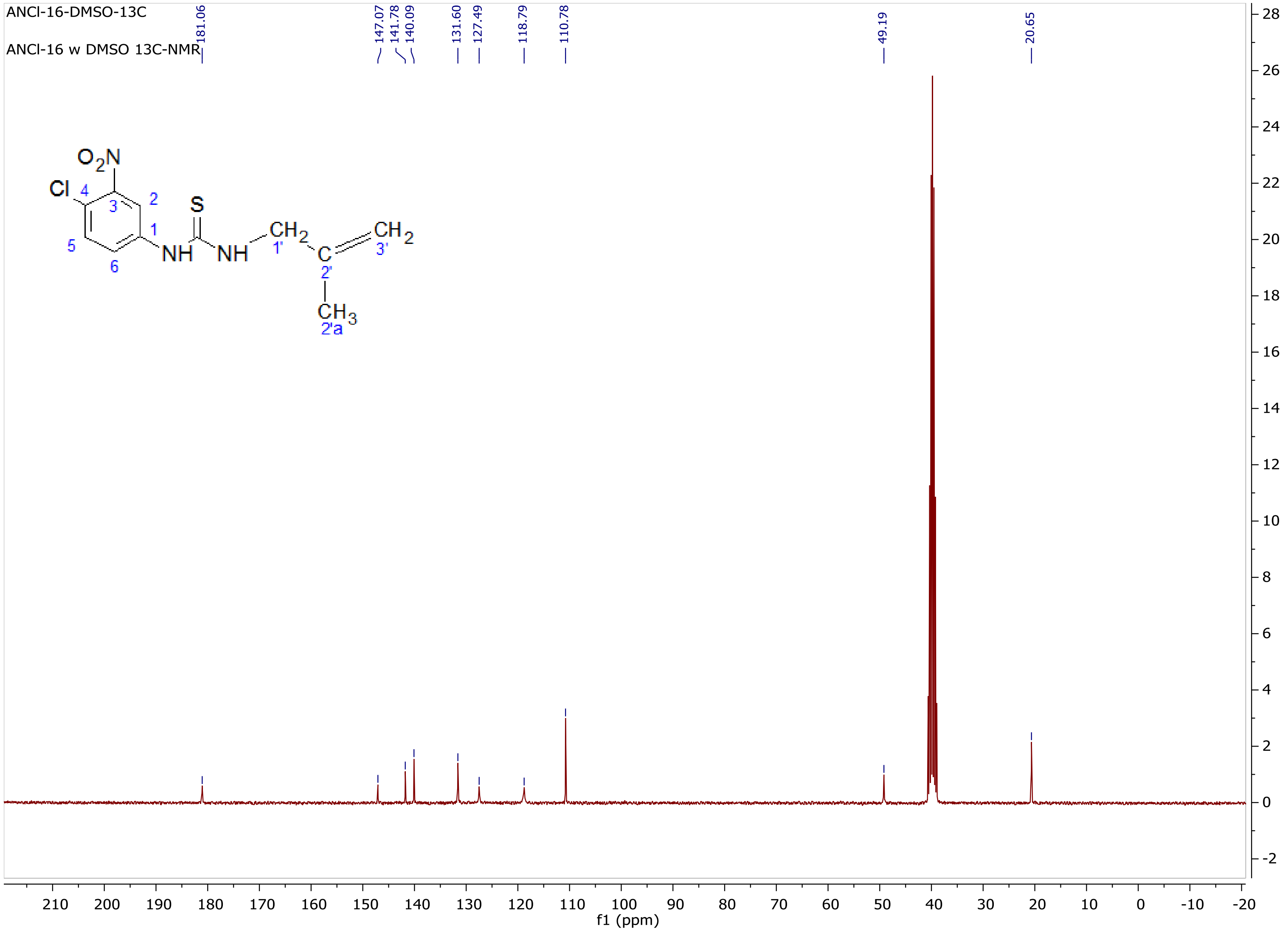

Spectrum 12. ^{13}C NMR of compd **27** (75.4 MHz, DMSO-d_6).

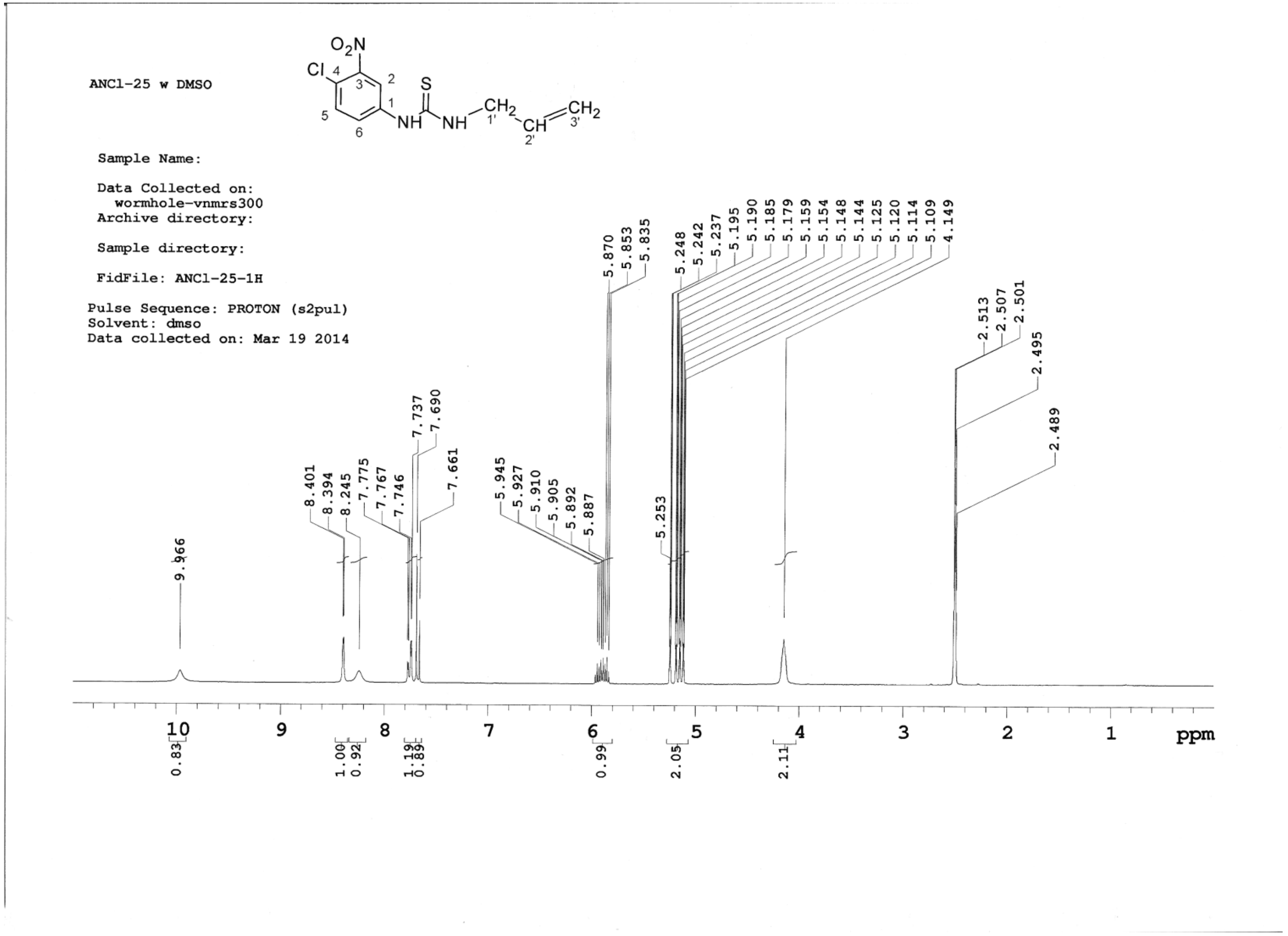

Spectrum 13. ^{1}H NMR of compd **28** (300 MHz, DMSO-d_6).

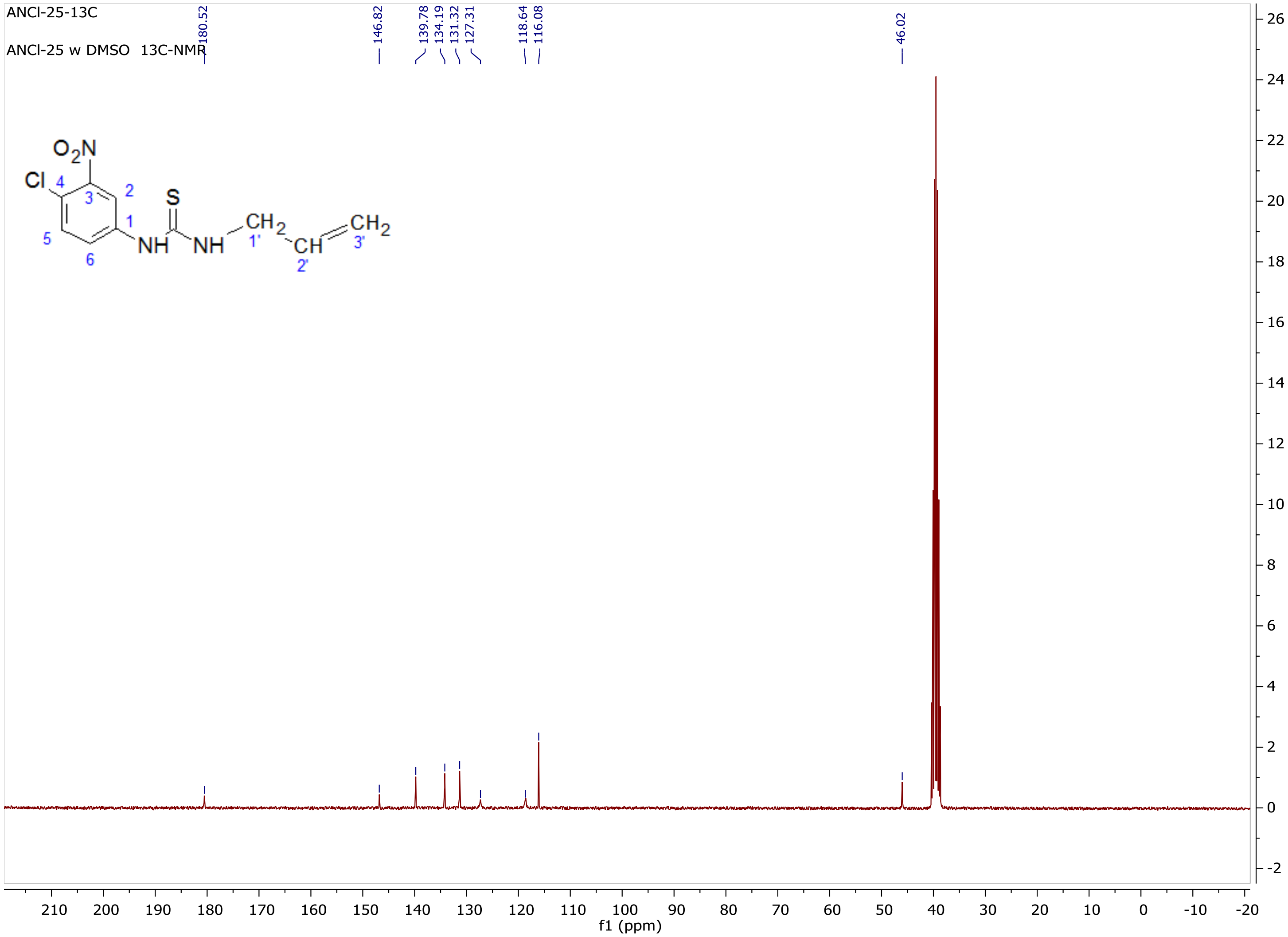

Spectrum 14. ^{13}C NMR of compd **28** (75.4 MHz, DMSO-d_6).

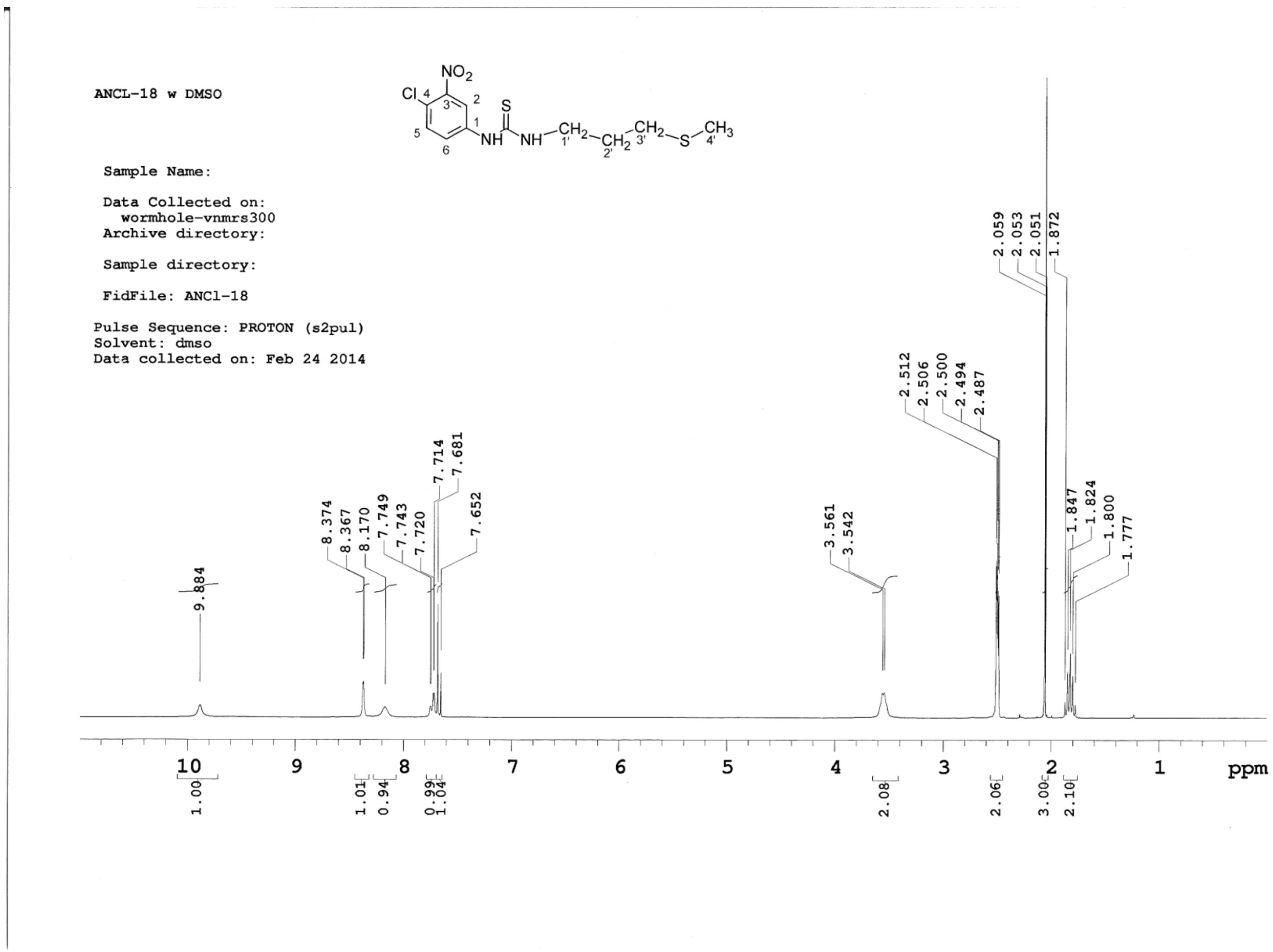

Spectrum 15. ^{1}H NMR of compd **29** (300 MHz, DMSO-d_6).

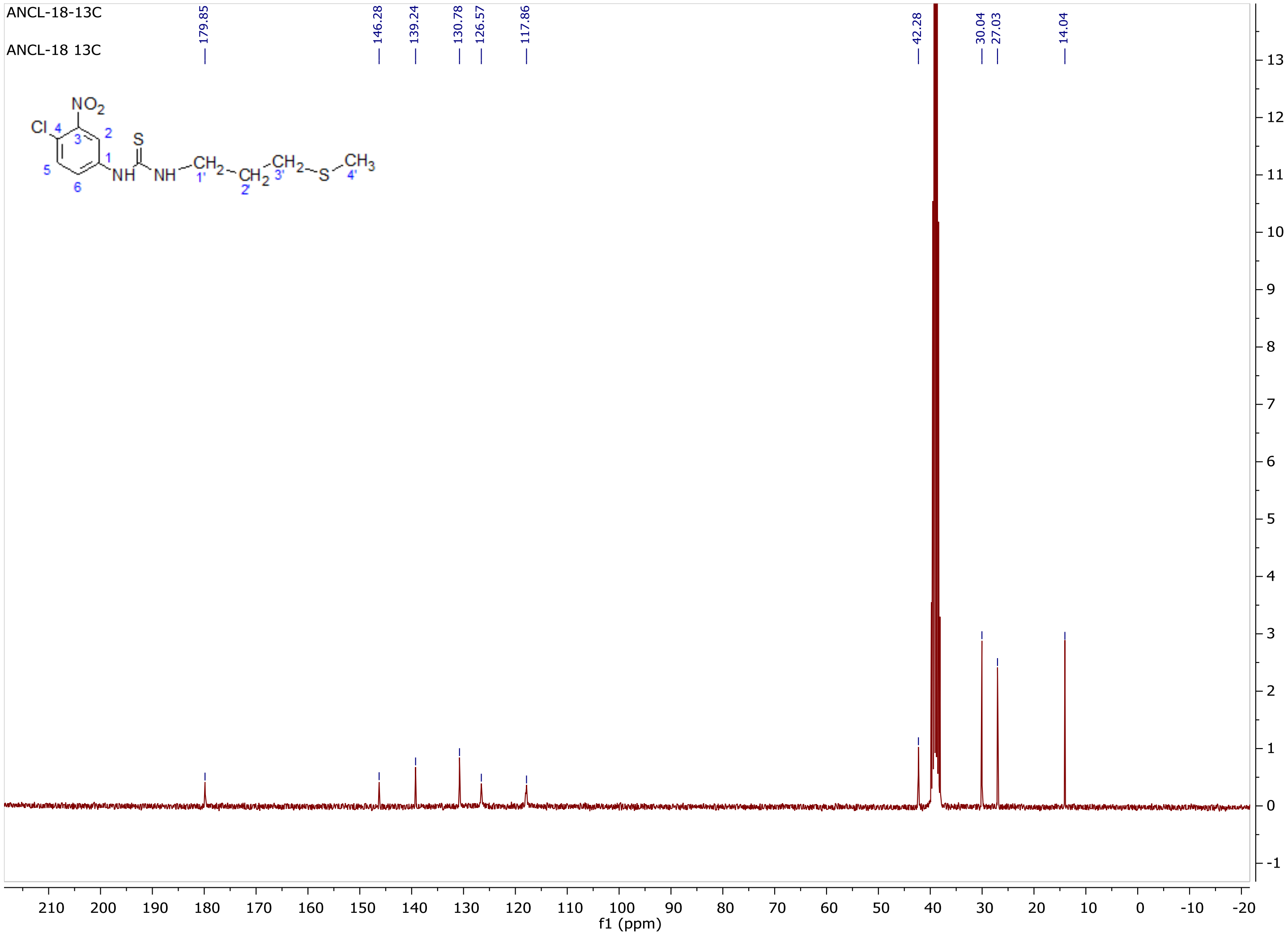

Spectrum 16. ^{13}C NMR of compd **29** (75.4 MHz, DMSO-d_6).

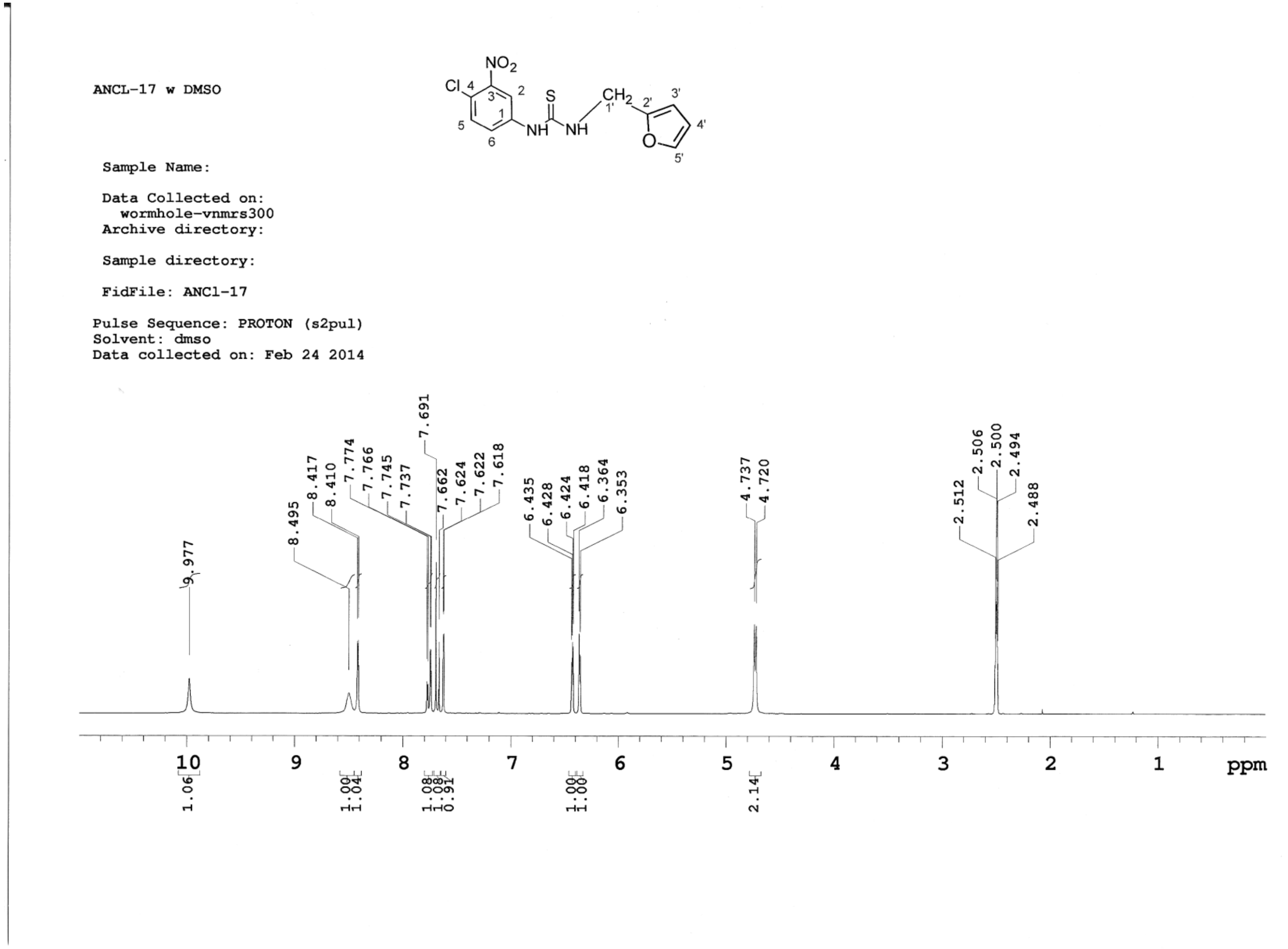

Spectrum 17. ^{1}H NMR of compd **30** (300 MHz, DMSO-d_6).

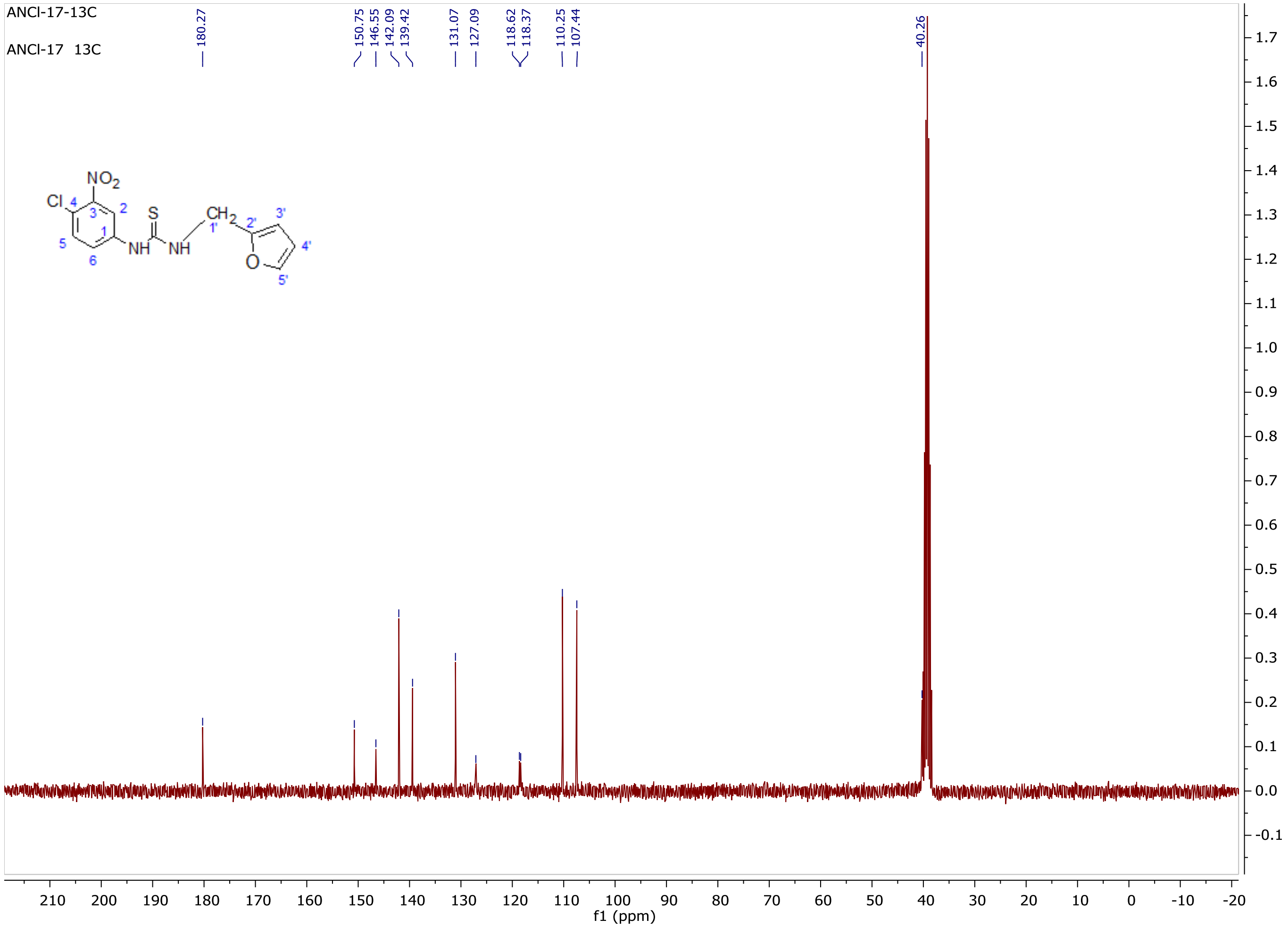

Spectrum 18. ^{13}C NMR of compd **30** (75.4 MHz, DMSO-d$_6$).
